# Identification and characterization of candidate detoxification genes in *Pharsalia antennata* Gahan (Coleoptera: Cerambycidae)

**DOI:** 10.3389/fphys.2022.1015793

**Published:** 2022-09-16

**Authors:** An-Jin Yang, Ning-Na Yin, Dan-Lu Chen, Yu-Ruo Guo, Yu-Jie Zhao, Nai-Yong Liu

**Affiliations:** ^1^ Key Laboratory of Forest Disaster Warning and Control of Yunnan Province, Southwest Forestry University, Kunming, China; ^2^ Key Laboratory of National Forestry and Grassland Administration on Biodiversity Conservation in Southwest China, Southwest Forestry University, Kunming, China

**Keywords:** *Pharsalia antennata*, cytochrome P450 monooxygenase, carboxylesterase, glutathione-S-transferase, detoxification, olfaction

## Abstract

The wood-boring beetles, including the majority of Cerambycidae, have developed the ability to metabolize a variety of toxic compounds derived from host plants and the surrounding environment. However, detoxification mechanisms underlying the evolutionary adaptation of a cerambycid beetle *Pharsalia antennata* to hosts and habitats are largely unexplored. Here, we characterized three key gene families in relation to detoxification (cytochrome P450 monooxygenases: P450s, carboxylesterases: COEs and glutathione-S-transferases: GSTs), by combinations of transcriptomics, gene identification, phylogenetics and expression profiles. Illumina sequencing generated 668,701,566 filtered reads in 12 tissues of *P. antennata*, summing to 100.28 gigabases data. From the transcriptome, 215 genes encoding 106 P450s, 77 COEs and 32 GSTs were identified, of which 107 relatives were differentially expressed genes. Of the identified 215 genes, a number of relatives showed the orthology to those in *Anoplophora glabripennis*, revealing 1:1 relationships in 94 phylogenetic clades. In the trees, *P. antennata* detoxification genes mainly clustered into one or two subfamilies, including 64 P450s in the CYP3 clan, 33 COEs in clade A, and 20 GSTs in Delta and Epsilon subclasses. Combining transcriptomic data and PCR approaches, the numbers of detoxification genes expressed in abdomens, antennae and legs were 188, 148 and 141, respectively. Notably, some genes exhibited significantly sex-biased levels in antennae or legs of both sexes. The findings provide valuable reference resources for further exploring xenobiotics metabolism and odorant detection in *P. antennata*.

## Introduction

The cerambycid beetles are members of the most diverse groups, with over 30,000 described extant species to date. The majority of cerambycid larvae feed on wood tissues of trees and thus are typical representatives of wood-boring pests (Slipinski et al., 2011). Similar to other herbivorous insects, each cerambycid beetle has evolved specialized feeding host plant(s). Therefore, they are most likely to develop various detoxification mechanisms to adapt the hosts. Apart from that, due to the differences of habitats, toxic compounds (like insecticides and herbicides) from the external environment further shape the evolution of detoxification-related enzyme genes, such as cytochrome P450 monooxygenases (P450s), carboxylesterases (COEs), and glutathione-S-transferases (GSTs) (Allison et al., 2004; Despres et al., 2007).

Insect P450 is the most extensively studied detoxification gene family, emphasizing the roles in insecticide resistance (Zhu et al., 2010; Zimmer et al., 2014; Dermauw et al., 2020). Beyond the degradation of P450s to synthetic insecticides, studies have demonstrated their involvement in detoxifying plant-derived toxic compounds (Amezian et al., 2022). Hence, this gene family is of paramount important for the adaption of herbivorous insects to host plants and habitats. For wood-boring pests including the majority of cerambycid beetles, they spend most of the life history in the trunk of trees. To defense plant secondary metabolites (often defensive compounds), some members of the P450 gene repertoire regulate their expression or generate many gene copies via gene duplications or alternative splicing, particularly for the CYP3 and CYP4 clans. In *Anoplophora glabripennis*, the expression of 25 P450 genes in guts was modulated after larvae fed on sugar maple (McKenna et al., 2016). Similarly, the genes of CYP4 and CYP9 clades in *Ips paraconfusus* were induced by feeding on the host plant *Pinus ponderosa* (Huber et al., 2007). Beyond the detoxification, P450 genes in *Ips pini* (*IpinCYP9T2*) and *Dendroctonus ponderosae* (*DponCYP345E2*) were associated with the pheromone production and odorant detection, respectively (Sandstrom et al., 2006; Keeling et al., 2013a).

The COE is another critical component of the insect defense system and is capable of hydrolyzing a variety of esters of carboxylic acids (Wheelock et al., 2005; Oakeshott et al., 2019). Similar to P450s, the transcription of COE genes is also induced by xenobiotic compounds, including host plant and man-made chemicals. In *A. glabripennis*, a number of COE genes had inducible expression in sugar maple-fed larvae, suggesting their putative roles in the detoxification of plant allelochemicals (McKenna et al., 2016). For the roles of COEs in metabolizing insecticides, several studies showed a particular focus on their resistance to organophosphates, including *Laodelphax striatellus* (Zhang et al., 2012), *Locusta migratoria manilensis* (Zhang et al., 2011), and *Nilaparvata lugens* (Small and Hemingway, 2000). In some cases, insect COEs specifically or highly expressed in antennae are responsible for the clearance of redundant plant odorants or sex pheromones, so as to recover the sensitivity of olfactory sensory neurons to semiochemicals (Durand et al., 2010; He et al., 2020; Wang et al., 2021).

In comparison to the above two gene families, the numbers of GSTs in most insects were relatively small, ranging from 28 genes in *A. glabripennis* to 43 in *Aethina tumida* for the Coleoptera, and 22 genes in *Plutella xylostella* to 45 in *Spodoptera litura* for the Lepidoptera (You et al., 2015; McKenna et al., 2016; Cheng et al., 2017; Evans et al., 2018; Zhao et al., 2020b). In the two other cerambycid beetles *Xylotrechus quadripes* and *Rhaphuma horsfieldi*, GST numbers identified from the transcriptomes were 18 and 20, respectively (Zhao et al., 2020b). As a multifunctional gene family presented ubiquitously in insects, GSTs, mainly members of insect-specific Delta and Epsilon classes, become well-known detoxification enzymes due to their involvement in insecticide resistance. Such the resistance of COEs to insecticides occurred in Coleoptera (Kostaropoulos et al., 2001), Lepidoptera (Liu et al., 2017), Diptera (Ranson et al., 2001) and Hemiptera (Zhou et al., 2013). Outside their association in insecticide resistance, GSTs also responded to plant defensive compounds as a positive feedback for adapting feeding host plants (Dai et al., 2016; Liu et al., 2021).

The cerambycid beetle, *Pharsalia antennata* Gahan (Coleoptera: Cerambycidae), is a wood-boring pest and feeds mainly on the Juglandaceae family. To date, information on this beetle is focused on its morphological characteristics, including the ultrastructure of sensilla on antennae and tarsi of both sexes (Yin et al., 2020). Like other cerambycids, *P. antennata* utilizes a detoxification system to adapt diverse host plants and complex habitats. Previously, the phase II enzyme uridine diphosphate (UDP)-glycosyltransferase (UGT) in *P. antennata* was surveyed, summing to a comparable number of 59 transcripts (Yin et al., 2022). Such the number raises the possibility that the other gene families in relation to detoxification may also have lineage-specific expansions in this beetle, deserving further studies. In the present study, we identified and characterized three other detoxification enzyme families (P450s, COEs and GSTs) in *P. antennata*, based on the transcriptomic data of 12 adult tissues. This study identifies candidate molecular targets for detoxifying toxic compounds and greatly improves the detoxification gene repertoires in *P. antennata*.

## Materials and methods

### Insects and tissue samples

*P. antennata* adults were collected from the trunks of *Juglans sigillata* at Santai Village, Dayao County, Chuxiong City, Yunnan Province (26°00′01.6″ N, 101°04′04.7″ E). The damaged walnut trees were cut and brought to the laboratory. Rearing conditions were described in a previous study (Yin et al., 2022). After adult emergence, female and male beetles were distinguished by sex, following morphological characteristics of abdomens and the lengths of antennae (Yin et al., 2020). To construct cDNA libraries of various tissues and investigate expression profiles of detoxification genes, we dissected 12 tissues from 3– to 5–days–old beetles of both sexes, including 10 antennae, five heads without antennae, three thoraxes, three abdomens, 10 legs and 15 wings per biological replicate. In the expression profiling analyses, three biological pools were collected. All the tissues were immediately frozen and then stored at –80°C.

### Total ribonucleic acid preparation and cDNA synthesis

Total RNA of each tissue was extracted using TRIzol reagent (Ambion, Life Technologies, Carlsbad, CA, United States), following the manufacturer’s instructions. Briefly, tissues were homogenized with 1 ml of TRIzol. We used a NanoDrop 1,000 Spectrophotometer (Thermo Fisher Scientific, San Jose, CA, United States) and agarose gel electrophoresis (1%, *w*/*v*) to measure the quality and concentration of purified RNA samples. Genomic DNA in RNA samples was digested with DNase I (Amplification Grade; Invitrogen Life Technologies, Carlsbad, CA, United States). For *de novo* transcriptome sequencing, at least 1 μg of total RNA for each tissue was sent to the Novogene Bioinformatics Technology Co. Ltd. (Tianjing, China). In the expression profiles of genes, cDNA synthesis was conducted with 1 μg of total RNA, according to the protocols of a PrimeScript RT reagent Kit with gDNA Eraser (TaKaRa, Dalian, Liaoning, China). Briefly, the reverse transcription reaction was performed at 37°C for 15 min, and stopped at 85°C for 5 s. Three biological templates for each tissue were prepared.

### Sequencing, assembly and annotation of the transcriptome

After assessing the quality and quantity of RNA samples by using a Qubit 2.0 Fluorometer (Invitrogen Life Technologies, Waltham, MA, United States) and an Agilent 2100 Bioanalyzer (Agilent Technologies, Santa Clara, CA, United States), cDNA libraries were produced via NEBNext® Ultra™ RNA Library Prep Kit for Illumina (NEB, United States), along with the manufacturer’s recommendations. The libraries were sequenced on an Illumina Hiseq–PE150 platform using a paired-end sequencing strategy. The initially generating sequences (raw reads) were filtered after removing adapter sequences, reads with poly-N and low quality reads. Next, the resulting reads (clean reads) were assembled into the trinity transcriptome using Trinity *v*2.5.1 (Grabherr et al., 2011), or used for subsequent analyses. After the transcripts were clustered by Corset *v*1.05 with default settings (Davidson and Oshlack, 2014), the longest transcript in each cluster was picked and pooled into a unigene database (defined as the unigene transcriptome). Using the unigenes as queries, we aligned them into seven functional databases (GO, Gene Ontology; KO, KEGG Ortholog; KOG/COG, EuKaryotic Orthologous Groups/Clusters of Orthologous Groups; NR, National Center for Biotechnology Information (NCBI) non-redundant (NR) protein sequences; NT, NCBI non-redundant nucleotide sequences; PFAM, Protein family and SwissProt). The annotated information of unigenes in each database was summarized.

### Estimation of gene expression levels

To obtain the expression levels of each gene in various tissues, clean reads from each tissue were mapped to the reference unigene transcriptome using Bowtie2 (Langmead and Salzberg, 2012). Next, reads of each gene in different tissues were counted by RSEM *v*1.2.15 (Li and Dewey, 2011). Expression levels of genes were calculated using a FPKM (fragments per kilobase of transcript per million mapped reads) method (Trapnell et al., 2010). For the analysis of differentially expressed genes (DEGs), the edgeR package (version 3.0.8) was used to adjust read counts via one scaling normalized factor (Robinson et al., 2010). Differential expression between two samples was determined using the DEGSeq R package (version 1.12.0) (Wang et al., 2009). The statistical significance of DEGs was set at a corrected *p* value <0.005 and |log_2_(foldchange)| >1.

### Identification of candidate detoxification genes

Considering that some short sequences were possibly lost during the process of Corset clusters, we queried two different transcriptomic data, i.e., the trinity transcriptome and the unigene transcriptome. A sequence query file was set using amino acid sequences of detoxification genes (P450s, COEs and GSTs) from three cerambycid beetles (*A. glabripennis*, *X. quadripes*, and *R. horsfieldi*) (McKenna et al., 2016; Zhao et al., 2020b) and a model coleopteran organism *Tribolium castaneum* (Oakeshott et al., 2010; Shi et al., 2012; Zhu et al., 2013). Three detoxification gene families in *P. antennata* were identified using amino acid sequences collected above as queries, by blasting the above two transcriptomes. The searching parameters were as follows: maximum hit of 20, BLOSUM62 matrix and a max *E*-value of 1e^−5^. The homology and conserved domains of detoxification enzymes were determined by blasting the NCBI NR protein sequence database. Further, we used identified genes as queries to iteratively search the transcriptomes until no novel candidate genes were found.

### Phylogenetics

Due to the size differences of three detoxification gene repertoires, we selected the corresponding protein sequences from different species to build the trees. In the P450 tree, P450 sequences from *A. glabripennis* (McKenna et al., 2016) and *P. antennata* were included. The COE tree was constructed with the protein sequences from *A. glabripennis* (McKenna et al., 2016), *P. antennta*, *T. castaneum* (Oakeshott et al., 2010) and *Drosophila melanogaster* (Claudianos et al., 2006). In the GST tree, seven coleopteran species were chosen, including *A. glab*ripennis (McKenna et al., 2016), *D. ponderosae* (Keeling et al., 2013b), *Leptinotarsa decemlineata* (Schoville et al., 2018), *R. horsfieldi*, *X. quadripes* (Zhao et al., 2020b), *P. antennata* and *T. castaneum* (Shi et al., 2012). The protein sequences were aligned using MAFFT *v*7.450 with the L–INS–I algorithm and default parameters (scoring matrix: BLOSUM62, offset value: 0.123 and gap open penalty: 1.53) (Katoh and Standley, 2013). The aligned sequences were used to infer maximum likelihood trees under the Whelan–And–Goldman (WAG) model with FastTree *v*2.1.11 (Price et al., 2010). The tree was visualized and edited using FigTree *v*1.4.4 (http://tree.bio.ed.ac.uk/software/figtree/).

### Expression profiling analysis

Based on the results by RNA sequencing (RNA–Seq), we randomly selected 48 *P450s*, 38 *COEs* and 15 *GSTs* to validate and investigate their expression in 12 tissues of both sexes. These genes were mainly composed of antenna-, thorax- or abdomen-enriched relatives. A reverse transcription PCR (RT–PCR) strategy was first employed to determine their tissue expression characteristics. To measure the quality and reliability of templates, we used a control gene in *P. antennata* (ribosomal protein S3, *PantRPS3*) to detect their usability (Yin et al., 2022). PCR reactions were conducted with a 25 μL mixture, containing 1 μL of cDNA, 1.5 μL of forward or reverse primers (10 μM). 2 μL of dNTP mixture (each 2.5 mM), 2.5 μL of 10×PCR buffer (Mg^2+^), 0.15 μL of TaKaRa Taq (5 U/μL) and 15.35 μL of sterile water. According to the manufacturer’s protocols of a TaKaRa Taq™ Kit (TaKaRa, Dalian, Liaoning, China), an annealing temperature of 55°C was applied with 35 cycles. If the results were inconsistent between RNA–Seq and RT–PCR, an additional replicate was performed using the third biological pool as the template. PCR products were analyzed using 1.2% (*w*/*v*) agarose gels. The primer sequences are listed in Supplementary Table S1.

To further determine the relative expression of detoxification genes in tissues and their putative roles in chemoreception, we picked 25 genes to examine their expression in antennae, legs and other tissues of female and male beetles using real-time quantitative PCR (qPCR). A reaction mixture of 20 μL was prepared, comprising 2 μL of cDNA, 10 μL of Bestar® SybrGreen qPCR mastermix (DBI® Bioscience, Germany), 0.5 μL of forward or reverse primers (10 μM), and 7 μL of sterile water. The specificity of PCR products was validated by melting curve analysis from 60 to 95°C. The amplification efficiencies of primers designed by Beacon Designer 8.14 (PREMIER Biosoft International, CA, United States) were checked using gradient dilutions of cDNA templates. The qPCR was run on qTOWER 2.2 (Analytic Jena AG, Germany) under the conditions: 2 min at 95°C, 40 cycles of 10 s at 95°C, 31 s at 58°C, and 30 s at 72°C. Fluorescence readings were taken at the elongation step of 72°C. Expression levels of detoxification genes were computed relative to two control genes (*PantRPS3* and ribosomal protein L10, *PantRPL10*), using a Q–Gene method (Muller et al., 2002; Simon, 2003). In order to ensure the reproducibility and reliability of qPCR data, the MIQE (Minimum Information for Publication of Quantitative Real-Time PCR Experiments) guidelines were followed (Bustin et al., 2009). Three biological templates for each tissue were used. The primers are listed in Supplementary Table S1. Significant differences between female and male tissues were compared by Student’s *t*–test in GraphPad Prism 7.00 (GraphPad Software Inc., San Diego, CA, United States).

## Results

### Transcriptome sequencing and assembly

**FIGURE 1:**
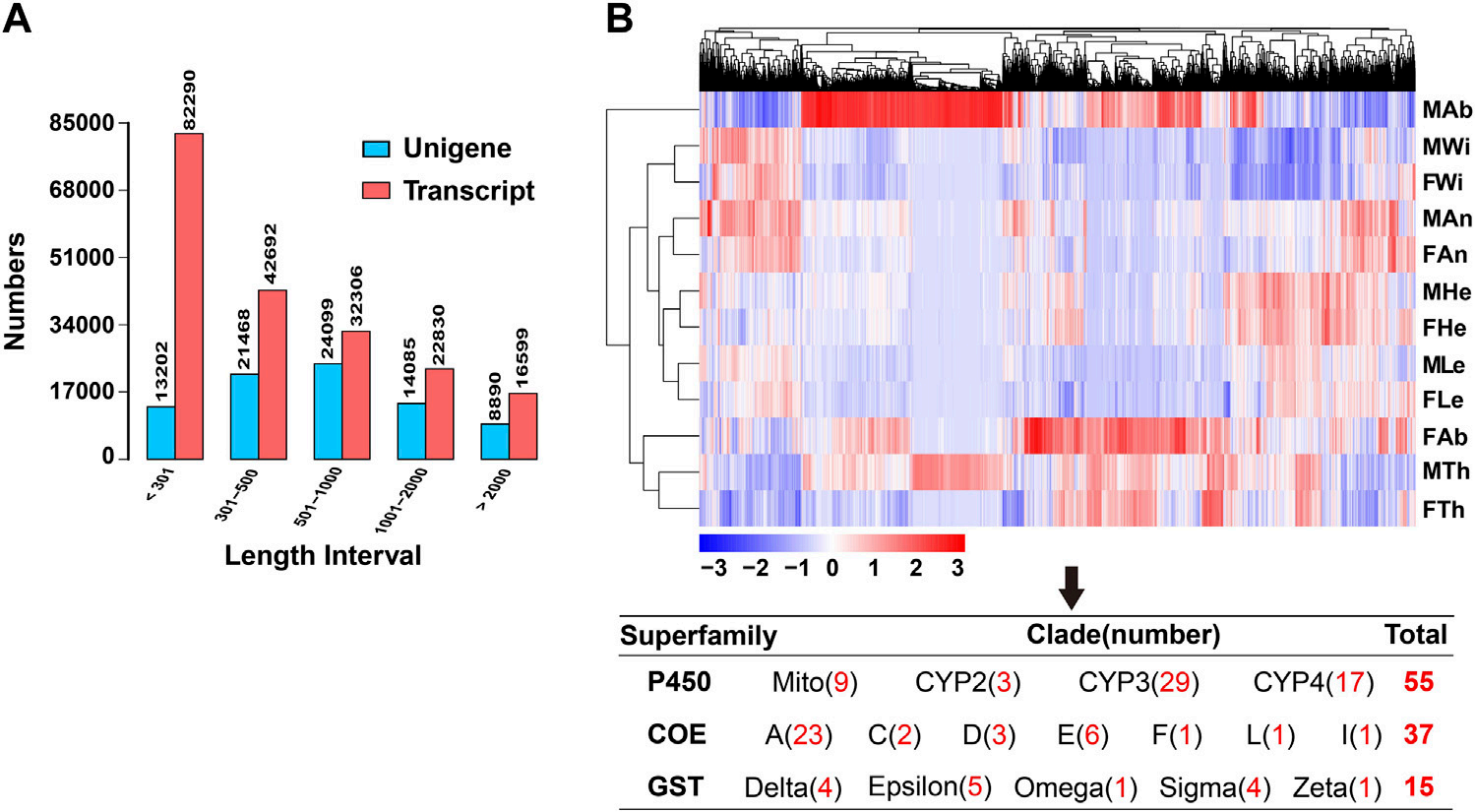
Summary of the transcriptome in *P. antennata*. **(A)** Length distribution and comparison of unigenes and transcripts. **(B)** The clustering analysis of differentially expressed genes. Differentially expressed detoxification genes (55 *P450s*, 37 *COEs* and 15 *GSTs*) in various clades are indicated.

Through the Illumina sequencing technology, we constructed and sequenced 12 cDNA libraries, representing 12 different tissues of female and male adults. In total, 669,610,108 raw reads were produced, comprising 327,705,854 sequences in males and 341,904,254 in females. All raw data of 12 tissues have been deposited in the NCBI Sequence Read Archive (SRA) database with the BioProject accession number PRJNA821002. The numbers of clean reads were variable in tissues, ranging from 44,598,376 sequences in male abdomens to 66,321,758 in female antennae. The clean data were summed to 100.28 gigabases (G), containing 668,701,566 sequences, high Q20 (97.36–97.90%) and Q30 (92.26–93.75%), and an average of 43.33% GC content (Table 1). All clean reads of 12 tissues were merged into a reference transcriptome (i.e., the unigene transcriptome), with a size of 76.9 megabytes (Mb). Such the assembly generated 196,717 transcripts and 81,744 unigenes, of which 41.83% (82,290) of the former were less than 301 bp in length and over half of unigenes (55.74%, 45,567) varied from 301 to 1,000 bp (Figure 1A).

**TABLE 1:** Summary statistics of the transcriptome in various tissues of *P. antennata*.

Sample	Raw read	Clean read	Size (G)	Q20 (%)	Q30 (%)	GC content (%)
MAn	50,076,080	50,029,846	7.50	97.80	93.41	43.80
MHe	52,416,128	52,331,828	7.84	97.64	93.00	40.92
MTh	51,504,424	51,447,100	7.72	97.56	92.74	38.97
MAb	44,654,408	44,598,376	6.68	97.81	93.34	41.98
MLe	63,106,660	63,008,978	9.46	97.72	93.30	44.59
MWi	65,948,154	65,847,588	9.88	97.81	93.52	48.52
FAn	66,426,828	66,321,758	9.94	97.79	93.41	45.09
FHe	56,476,494	56,396,500	8.46	97.82	93.43	41.54
FTh	52,716,586	52,647,352	7.90	97.36	92.26	38.74
FAb	58,095,240	58,025,160	8.70	97.90	93.62	42.26
FLe	56,326,912	56,259,356	8.44	97.69	93.20	43.91
FWi	51,862,194	51,787,724	7.76	97.90	93.75	49.70
Total/Mean	669,610,108	668,701,566	100.28	97.73	93.25	43.33

MAn, male antennae; MHe, male heads (removing antennae); MTh, male thoraxes; MAb, male abdomens; MLe, male legs; MWi, male wings; FAn, female antennae; FHe, female heads (removing antennae); FTh, female thoraxes; FAb, female abdomens; FLe, female legs and FWi, female wings.

### Transcriptome analysis and gene functional annotation

A comparative analysis of unigenes among 12 tissues revealed 7,998 DEGs, accounting for 9.78% of all the unigenes. Clustering analyses showed that the majority of DEGs were enriched in female and male abdomens. In contrast, a number of DEGs were found to have relatively low expression in wings of both sexes. Among these DEGs, a comparable number of differentially expressed detoxification genes were identified, including 55 *P450s*, 37 *COEs* and 15 *GSTs*. Of these, CYP3 (29 DEGs) and CYP4 (17 DEGs) members contributed to the majority of differentially expressed P450s, differential COEs mainly presented in clade A (23 DEGs) and differential GSTs mainly presented in Epsilon (five DEGs), Delta (four DEGs) and Sigma (four DEGs) subclasses (Figure 1B).

**FIGURE 2:**
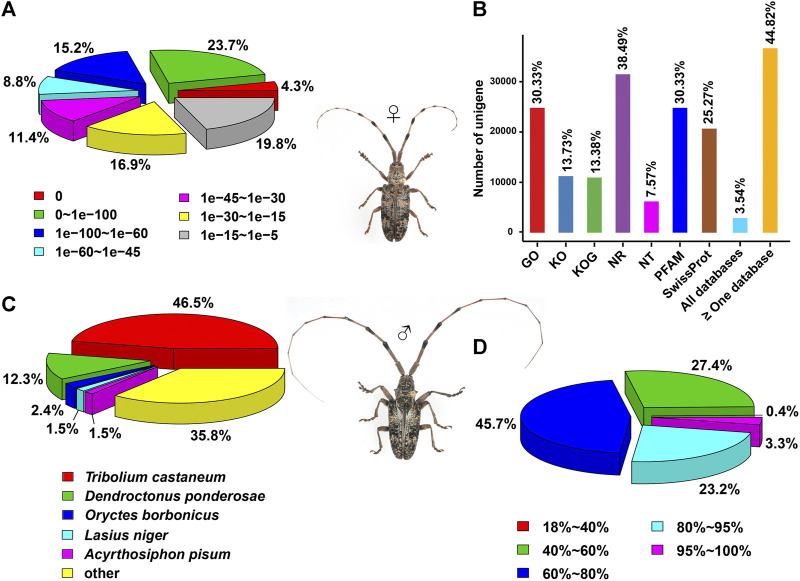
Annotation of the transcriptome in *P. antennata* illustrating the *E*-value distribution **(A)**, database annotation **(B)**, species classification **(C)**, and similarity distribution **(D)**.

Out of the 81,744 unigenes, most of them were distributed in two regions of *E*-values by blasting the genes against the NCBI NR protein sequence database, accounting for 38.9% of the genes for 0–1e^−60^ and 36.7% for 1e^−30^–1e^−5^ (Figure 2A). Further, 38.49% (31,471), 30.33% (24,795), 30.33% (24,795) and 25.27% (20,663) of the genes were annotated separately in NR, GO, PFAM and SwissProt databases, representing the most abundant presence among seven databases. The genes annotated in at least one database contributed to 44.82% (36,643) of the unigenes, but only 3.54% (2,901) of the genes were mapped into all seven databases (Figure 2B). In the NR annotation, *P. antennata* unigenes were mainly aligned to those in *T. castaneum*, accounting for 46.5% of the genes (Figure 2C). Approximately 72.2% of unigenes in *P. antennata* shared more than 60% similarities with those in the NCBI NR protein sequence database (Figure 2D).

### Identification of candidate genes involving detoxification

Based on the trinity transcriptome and the unigene transcriptome, a blasted-based homology search led to the identification of a total of 215 transcripts encoding 106 P450s, 77 COEs, and 32 GSTs. The P450 sequences in *P. antennata* were sent to the Cytochrome P450 Nomenclature Committees for the nomenclature. COEs were numbered 1 to 77 (*COE1*–*COE77*), and GST names were assigned with a combination of a lowercase of the GST subclass and numbers (like *GSTe1*). Four P450s (*CYP307B1*/*CYP6BJ9*/*CYP6BJ67*/*CYP9Z200*) and four COEs (*COE2*/*COE60*/*COE74*/*COE76*) were absent in the unigene transcriptome. The remaining 207 detoxification genes were presented in two transcriptomes. Of the identified genes, 129 relatives were predicted to have complete open reading frames (ORFs), with the protein sizes of 427–591 amino acids for 63 full-length *P450s*, 401–1,292 amino acids for 41 full-length *COEs*, and 204–244 amino acids for 25 full-length *GSTs*. Other 86 genes were partial sequences, including 43 *P450s* (99–542 amino acids), 36 *COEs* (106–554 amino acids) and seven *GSTs* (114–190 amino acids) (Supplementary Table S2). All identified genes have been submitted to the NCBI GenBank database, under the accession numbers OP314595–OP314809.

In the analyses of NCBI blast hits, approximately 90% (193/215) of detoxification genes in *P. antennata* were aligned to the corresponding genes from *A. glabripennis*, with relatively high conservation (>50% amino acid identities). In particular, a comparable number of genes (100/193) shared over 80% amino acid identities with their respective orthologs in *A. glabripennis* (Supplementary Table S2).

### Phylogeny and expression profile of candidate cytochrome P450 monooxygenase genes

**FIGURE 3:**
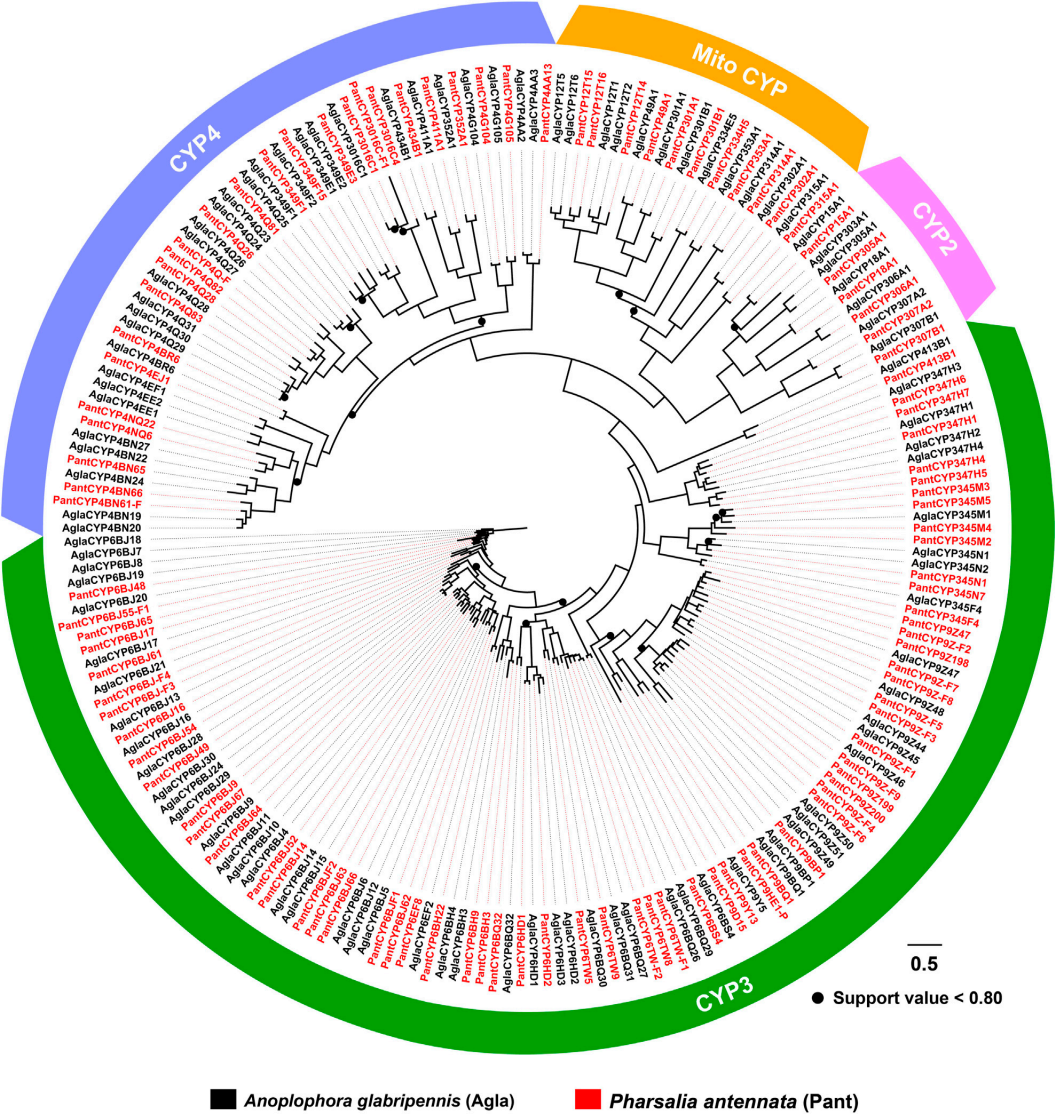
Maximum-likelihood tree of P450s in two cerambycid beetles. The P450 proteins in *P. antennata* are highlighted in red.

With an aligned protein sequence of 104 *A. glabripennis* P450s and 106 *P. antennata* P450s, a maximum likelihood tree was inferred. The phylogeny classified cerambycid P450s into four clades, representing 11 mitochondrial (Mito) members, six CYP2s, 64 CYP3s and 25 CYP4s in *P. antennata*. Between *P. antennata* and *A. glabripennis*, the former harbored more members in the CYP3 clade, but relatively fewer CYP4s compared to *A. glabripennis* (CYP3: 55 genes; CYP4: 31 genes). Other two clades Mito and CYP2 had similar gene numbers between the two cerambycid beetles. The tree revealed 52 orthologous pairs between the two species, showing 1:1 relationships in eight Mito, six CYP2, 25 CYP3 and 13 CYP4 pairs. Hence, in most cases, P450s between the beetles shared a high degree of conservation. Exceptionally, we detected a small cluster in *P. antennata*, representing PantCYP9Z-F4/CYP9Z-F6/CYP9Z-F9/CYP9Z199/CYP9Z200 in the CYP3 clan (Figure 3).

Based on FPKM values of RNA–Seq, 98 out of 102 *P450* genes had detectable expression in at least one tissue (FPKM >1). The majority of the genes displayed a broad tissue expression profile, some of which were enriched in each tissue such as *CYP12T14* in Mito, *CYP18A1* in CYP2, *CYP347H1*/*CYP6HD1*/*CYP9Z198* in CYP3, and *CYP4G104*/*CYP4Q82*/*CYP4Q83* in CYP4. Four genes had extremely low levels in tissues (0 < FPKM <1), including *CYP307A2*/*CYP9Z-F6*/*CYP9Z-F7*/*CYP434B1*. In antennae and abdomens, there were 80 and 91 genes, respectively, with FPKM values of above 1. Of these, some genes appeared to have obviously sex-biased levels in the two tissues. For instance, *CYP4Q28* showed particularly high expression in male antennae, with a 464.98–fold difference relative to females. *CYP334H5* was a male-abdomen-dominant gene, showing 86.25-fold higher expression than females (Supplementary Table S3).

**FIGURE 4:**
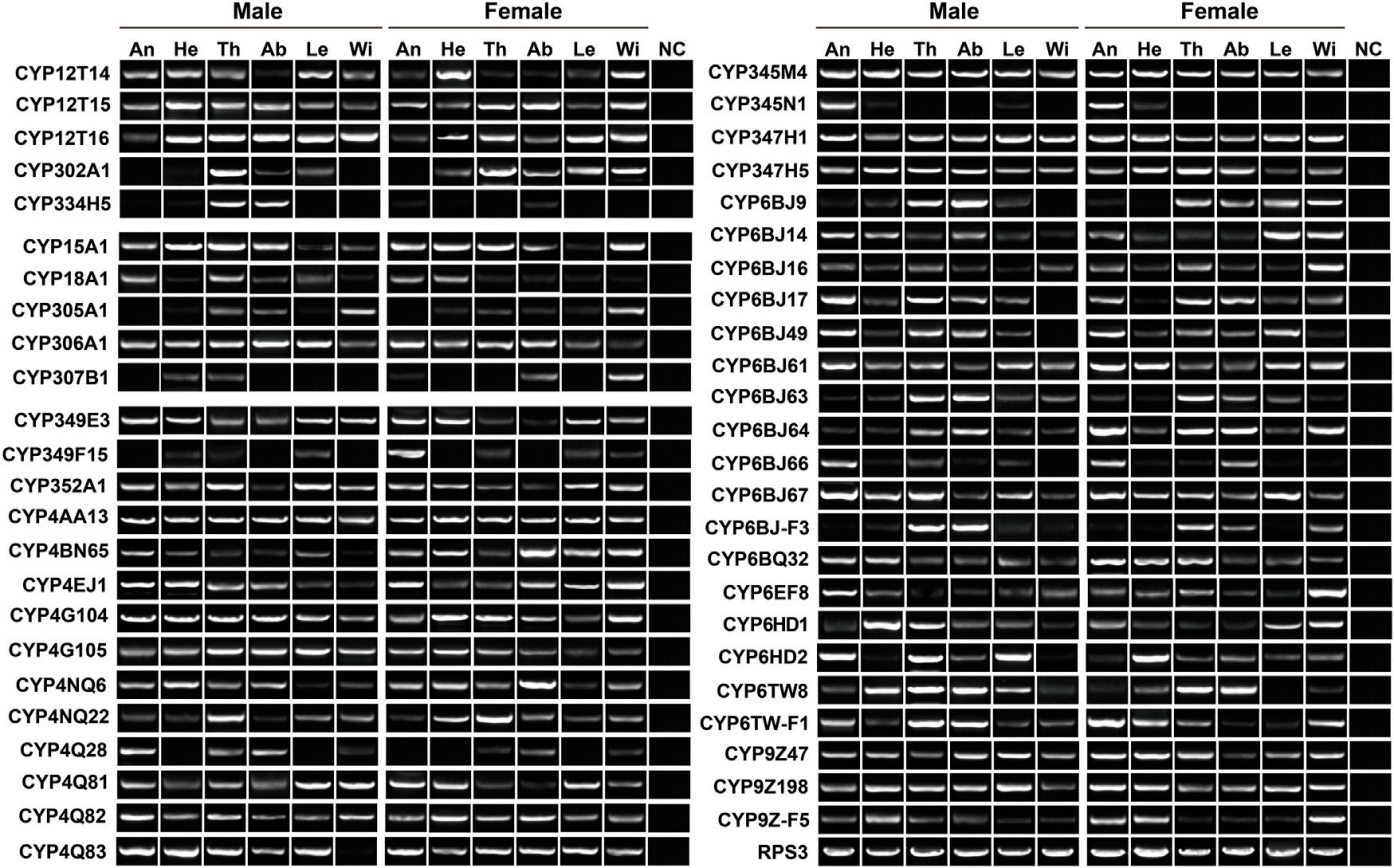
Expression pattern of 48 *P450* genes in various tissues of *P. antennata*. The abbreviation of tissues is seen in Table 1. NC, negative control using sterile water as the templates.

To validate the expression of P450 genes in tissues, we mainly focused on the genes highly expressed in antennae, thoraxes or abdomens, and further investigated their tissue presence by RT–PCR. Accordingly, a total of 48 relatives were selected, representing five Mitos, five CYP2s, 24 CYP3s and 14 CYP4s. In agreement with RNA–Seq results, most of the genes were widely transcribed in various tissues. As shown in RT–PCR assays, as many as 31 genes were presented in all 12 tissues, including three, two, 10 and 16 genes in Mito, CYP2, CYP3 and CYP4 clans, respectively. Notably, molecular evidence of *CYP4Q28* and *CYP334H5* further supported their preferential expression in male antennae and male abdomens, respectively. In addition, the expression of *CYP349F15* and *CYP6BJ64* was female-antenna-biased, whereas *CYP6HD2* had a stronger band in male antennae compared to females. *CYP345N1* exhibited the highest expression in antennae of both sexes among 12 tissues (Figure 4).

### Phylogeny and expression profile of candidate carboxylesterase genes

**FIGURE 5:**
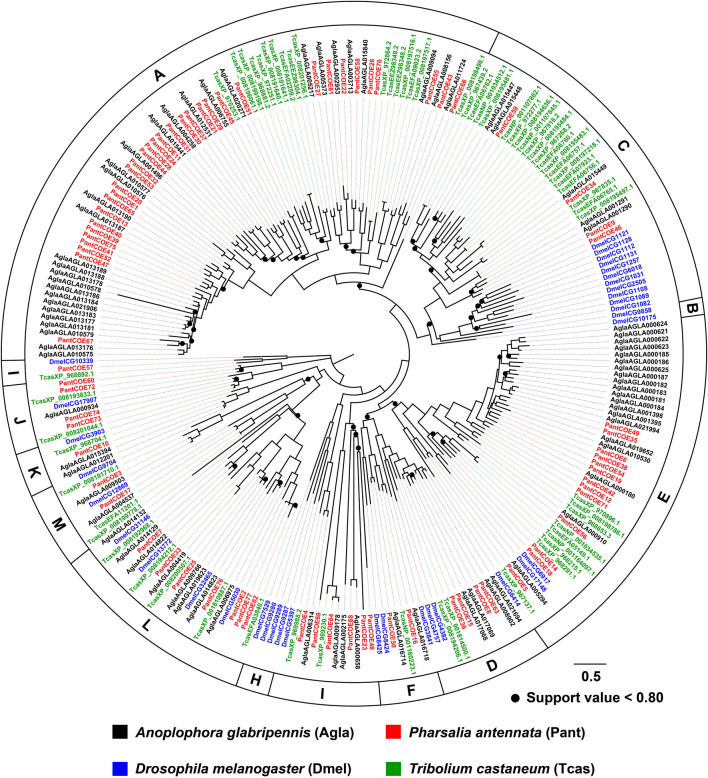
Maximum-likelihood tree of COEs in three coleopteran species and *D. melanogaster*. The P450 proteins in *P. antennata* are highlighted in red.

The phylogenetic tree built by 79 AglaCOEs, 77 PantCOEs, 63 TcasCOEs and 35 DmelCOEs revealed 10 clades in Coleoptera (clades A–F/H–M), excluding clades B and H containing COEs solely from *D. melanogaster*. *P. antennata* COEs were distributed in each of 10 clades, with the majority of clade A (33 relatives). Other nine clades were composed of relatively few genes, ranging from one to 13 members. Similar to *A. glabripennis*, *P. antennata* possessed two lineage-specific expansions in clades A and E, i.e., COE39–COE41/COE47/COE52/COE75 and COE6/COE19/COE35/COE38/COE49/COE54. Between *A. glabripennis* and *P. antennata*, there were 31 orthologous pairs dispersed into eight clades, except for clades D and K. Among three coleopteran species, two cerambycid beetles had a similar proportion of genes in each clade, with the exception of clade E where a large species-specific expansion (15 genes) was observed in *A. glabripennis*. In comparison, *T. castaneum* had contracted genes in clade A (17 genes), but an obvious expansion was observed in clade C (20 genes) (Figure 5).

Except for *COE2*/*COE60*/*COE74*/*COE76*, the remaining 73 *COEs* had available FPKM values in tissues. However, four genes had an extremely low expression in tested tissues (0 < FPKM <1), including *COE61*/*COE62*/*COE70*/*COE73*. Three COE genes (*COE15*/*COE34*/*COE64*) were broadly expressed in each tissue at a high level (FPKM >10). The numbers of COEs presented in antennae, thoraxes and abdomens (FPKM >1) were 32, 60 and 63, respectively. Of the tested tissues, some genes were preferentially transcribed in males or females with over 10–fold differences. For instance, *COE20* and *COE51* separately displayed 22.17– and 17.11–fold higher expression in female thoraxes than males. In contrast, the expression level of *COE67* in male thoraxes was 10.46–fold higher relative to females. Such the sex-biased expression was also obtained in abdominal tissues, including *COE51* (17.63–fold higher) and *COE53* (38.85–fold higher) in female abdomens, as well as *COE56* in males (140.20–fold higher) (Supplementary Table S3).

**FIGURE 6:**
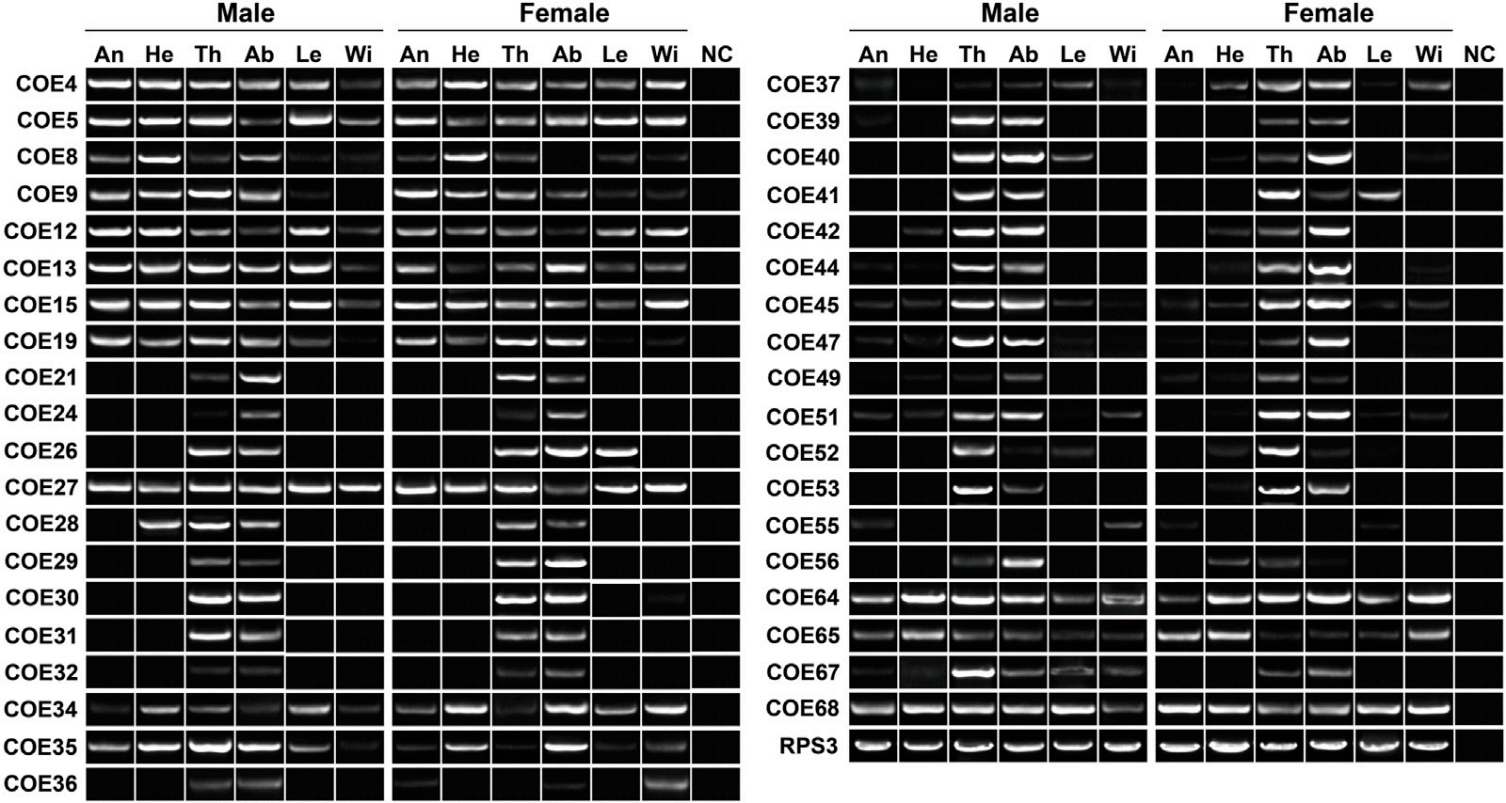
Expression pattern of 38 *COE* genes in various tissues of *P. antennata*. The abbreviation of tissues is seen in Table 1. NC, negative control using sterile water as the templates.

RT–PCR was employed to further check the expression of 38 *COEs* highly transcribed in antennae, thoraxes or abdomens. Outside *COE55*, the remaining 37 genes were detected in thoraxes or abdomens. In the antennae, 18 *COEs* were found to have the expression. Overall, molecular results with RT–PCR assays well support the existence of 38 COEs in tissues (Figure 6).

### Phylogeny and expression profile of candidate glutathione-S-transferase genes

**FIGURE 7:**
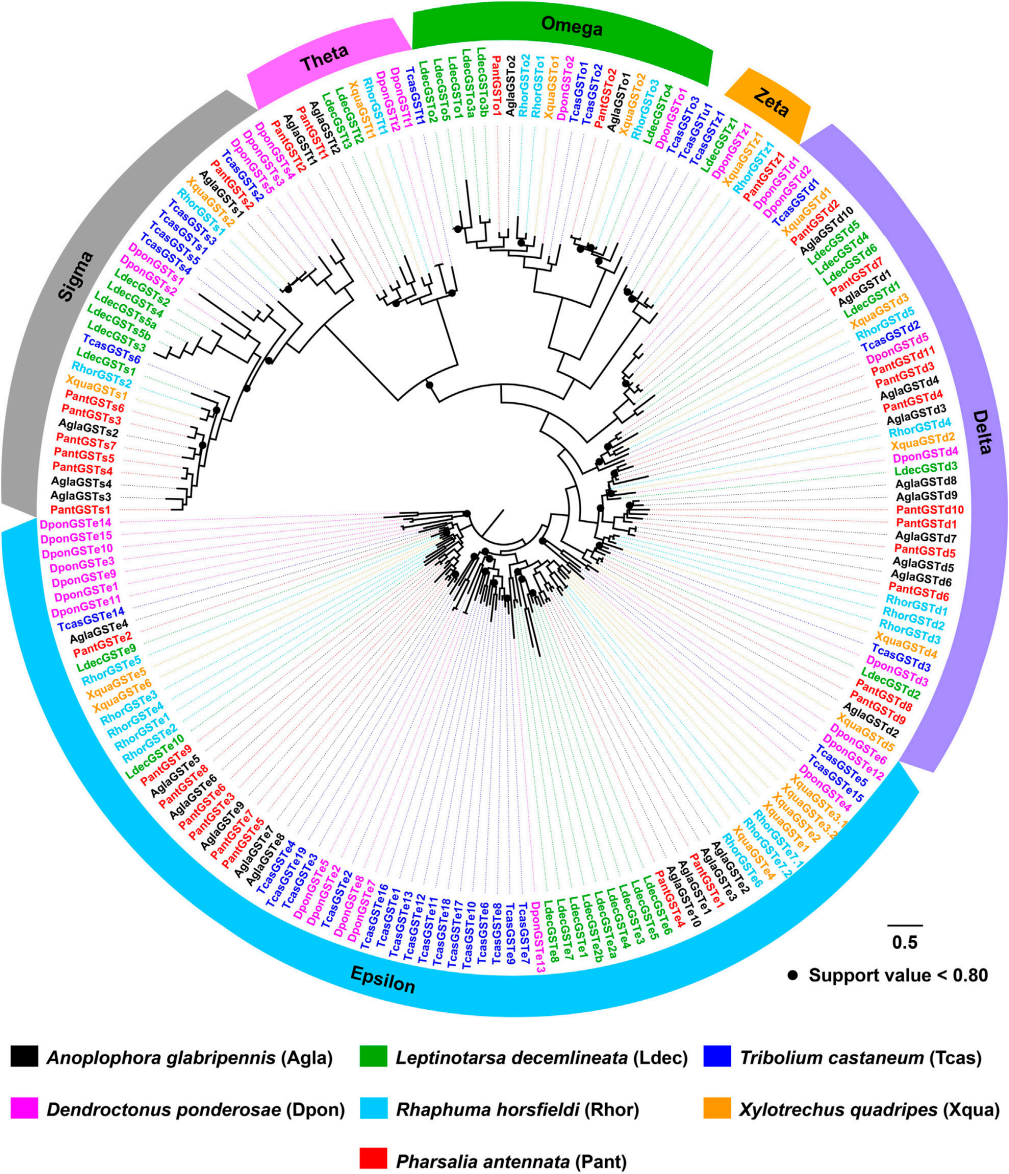
Maximum-likelihood tree of GSTs in seven coleopteran species. The GST proteins in *P. antennata* are highlighted in red.

The tree of 194 GSTs from seven coleopteran species was inferred, including 98 sequences in four cerambycids *A. glabripennis*, *P. antennata*, *R. horsfieldi* and *X. quadripes*. In six subclasses, the Delta clade comprised the largest number of *P. antennata* GSTs (11 relatives), followed by Epsilon (nine relatives) and Sigma (seven relatives) clades. This feature on the largest proportion of GSTs in Delta and Epsilon subclasses was observed in the other three cerambycid beetles. As indicated in the tree, non-cerambycid species harbored at least one species-specific cluster. Differing from that, GSTs from the longhorned beetles mainly clustered together in Cerambycidae-specific patterns. Among four cerambycids, only two orthologous groups were detected, representing AglaGSTo1/PantGSTo2/RhorGSTo3/XquaGSTo2 and AglaGSTs1/PantGSTs2/RhorGSTs1/XquaGSTs2. Between *A. glabripennis* and *P. antennata*, there were 1:1 orthologs in 11 clades distributed into five GST subclasses outside Zeta (Figure 7).

**FIGURE 8:**
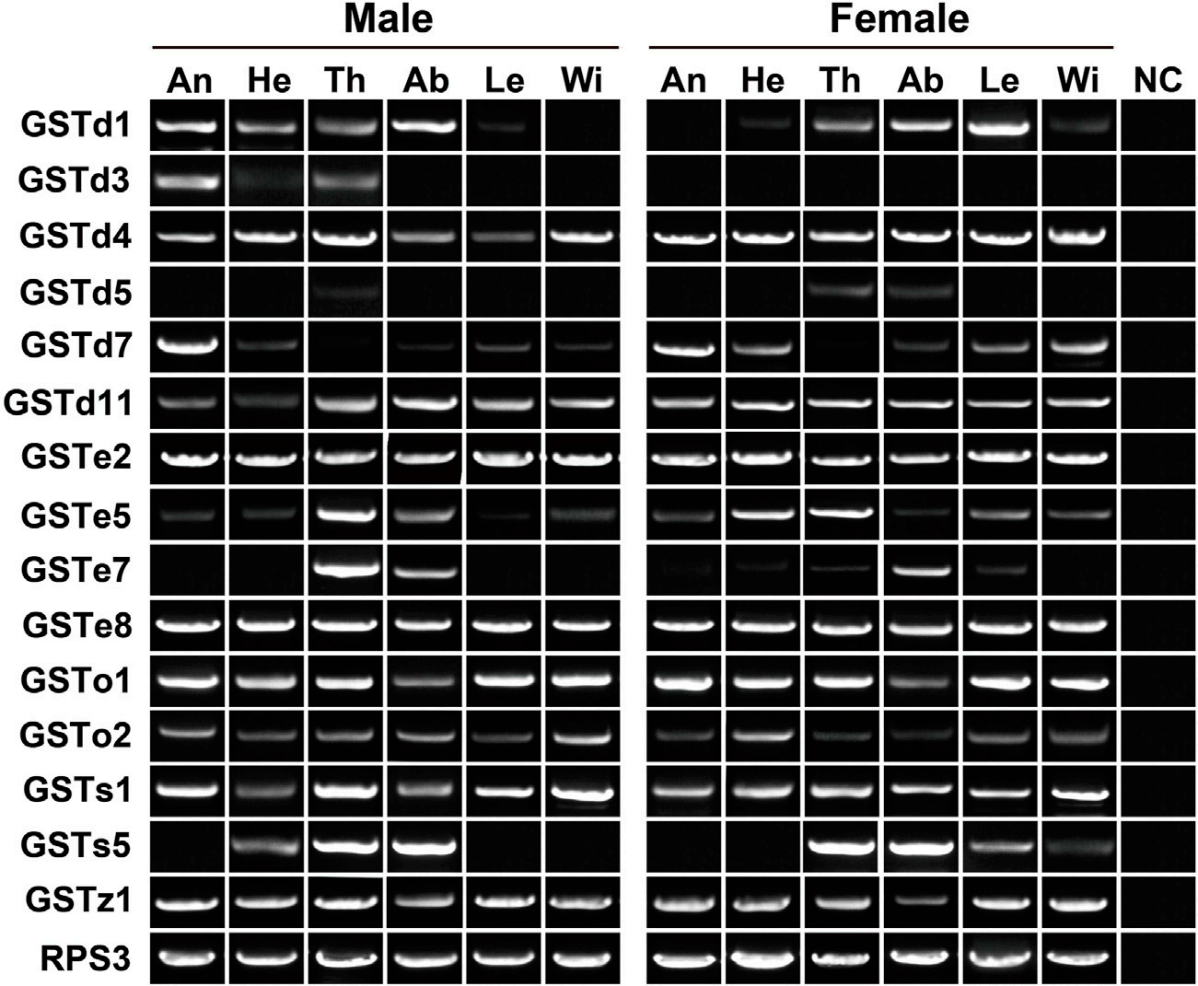
Expression patterns of 15 *GST* genes in various tissues of *P. antennata*. The abbreviation of tissues is seen in Table 1. NC, negative control using sterile water as the templates.

In the expression profiling analyses of *GSTs*, all 32 genes had the expression in at least one tissue, with FPKM values of above 1. In particular, nine genes were enriched in all 12 tissues (FPKM >10). Following the standard of FPKM >1, we identified 24, 29 and 30 *GSTs* in antennae, abdomens and thoraxes, respectively. A Delta gene, *GSTd2*, was highly expressed in female abdomens with a 10.88–fold difference relative to males. The *GSTe3* expression was restricted to female abdomens and thoraxes (Supplementary Table S3). In RT–PCR analyses of 15 *GSTs*, eight genes exhibited a wide tissue expression, consistent with RNA–Seq results. Except for *GSTd5*/*GSTe7*/*GSTs5*, other 12 genes were detected in antennae. Nearly all the genes had the expression in thoraxes (14 relatives) or abdomens (14 relatives) (Figure 8).

### Candidate detoxification genes involving chemoreception

**FIGURE 9:**
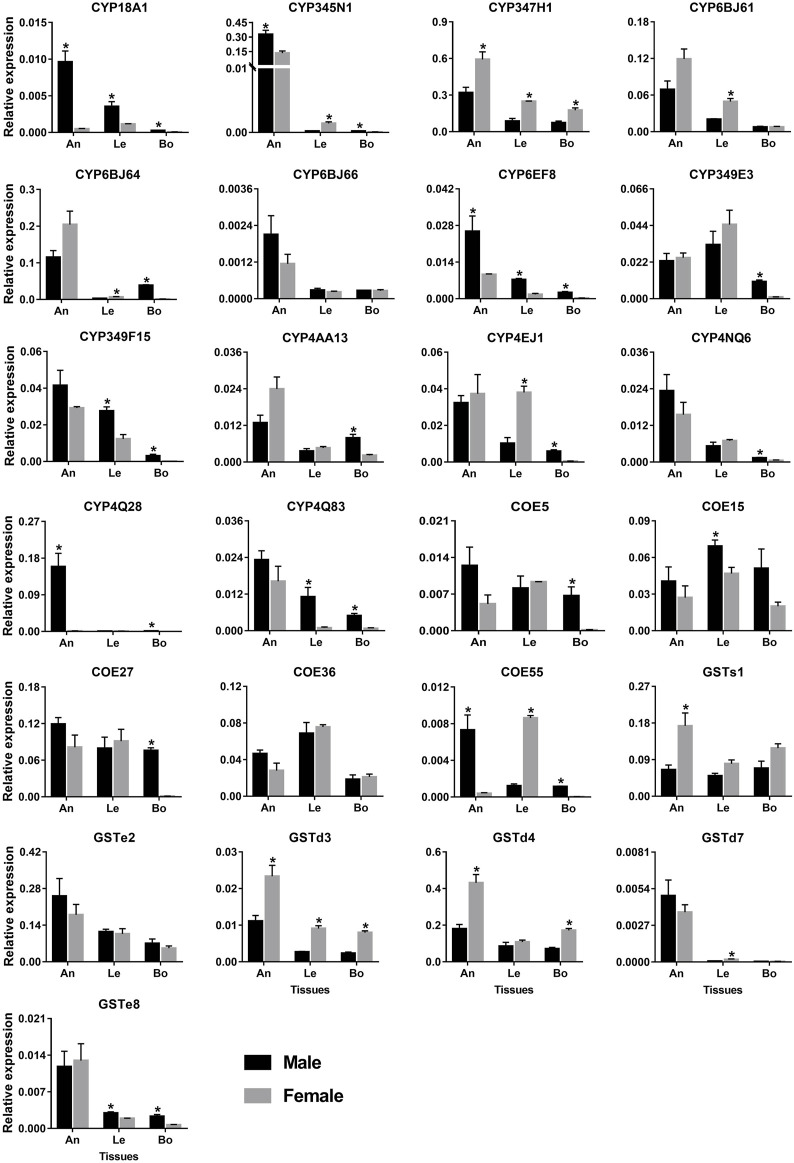
qPCR analysis of 25 detoxification genes in various tissues of *P. antennata*. Data denote mean ± standard errors. Asterisks represent significant differences between male and female tissues (ANOVA, Student’s *t*–test, *p* < 0.05). An, antennae; Le, legs and Bo, bodies without antennae and legs.

The antenna and leg are important chemosensory organs in insects, mainly serving as smell and taste (Hansson and Stensmyr, 2011). Combining the results of RNA–Seq (FPKM >1) and RT–PCR, 148 and 141 out of 215 genes had the expression in antennae and legs, respectively, representing 87 and 77 *P450s*, 36 and 38 *COEs*, and 25 and 26 *GSTs*. Here, we further used qPCR to investigate the relative expression of 25 genes specifically or highly expressed in the two chemosensory tissues. As a result, all 25 genes could be detected in the antennae with the majority of them being antenna-enriched relatives. Of 14 *P450* genes, 12 relatives were enriched in the antennae, of which four genes (*CYP18A1*/*CYP345N1*/*CYP6EF8*/*CYP4Q28*) were significantly male-biased relatives while *CYP347H1* showed a significant level in females. Notably, *CYP4Q28* displayed a 159.64–fold higher expression in male antennae compared to females, further supporting RNA–Seq and RT–PCR results. Some *P450* genes, like *CYP349E3*/*CYP349F15*/*CYP4EJ1*, showed relatively high expression in legs. However, most *P450s* had relatively low expression in bodies (removing antennae and legs). A significantly sex-biased expression was also obtained in legs and bodies, such as four *P450s* in male legs, five in female legs, 11 in male bodies and one in female bodies (Figure 9).

Five *COE* genes were abundantly expressed in antennae and legs. Of these, the expression of *COE15* was significantly higher in male legs than females, whereas *COE55* was a female-leg-biased gene. Besides, *COE55* also showed a significant level in male antennae. Similar to *P450s*, *GST* genes were expressed in the antennae at a high level. Three *GST* genes (*GSTd3*/*GSTd4*/*GSTs1*) exhibited significantly higher expression in female antennae compared to males. In legs, *GSTd3* was a female-biased gene while *GSTe8* was significantly enriched in males (Figure 9).

## Discussion

The wood-boring beetles, including *P. antennata* of the Cerambycidae family, have developed complex detoxification mechanisms to adapt specialized host plants (Allison et al., 2004; McKenna et al., 2016). Considering the differences of host plants, as well as special habitats underneath the bark for wood-borers, it is hypothesized that these beetles, particularly for generalists, are possibly subjected to the defences of more plant secondary metabolites. Such the defenses of plants to herbivorous insects may drive the expansions of P450s, COEs or GSTs via gene duplications or alternative splicing in insects. As implied in Coleoptera, the generalists (polyphagous species) in general possessed more P450s and COEs compared to the specialists (stenophagous and oligophagous species) (Zhao et al., 2020b). Correspondingly, a large size of the detoxification enzyme gene repertoire, to some extent, possibly reflects the host plant range of one coleopteran species. Our study identified a comparable number of *P450s* (106 genes) and *COEs* (77 genes) from the *P. antennata* transcriptome. Although host plants of this beetle remain to be largely unknown to date, relatively large detoxification gene repertoires are regarded as an implication on a broad range of host plants in *P. antennata*.

Among four cerambycid beetles *P. antennata*, *A. glabripennis*, *R. horsfieldi* and *X. quadripes*, the Asian longhorned beetle *A. glabripennis* was the only species with available genome sequences (accession number: GCA_000390,285.2) (McKenna et al., 2016). In comparison, this beetle harbored more P450s (125 genes) and COEs (107 genes), as well as nearly equal GST numbers (28 genes) with *P. antennata*. This result indicated that some genes encoding P450s and COEs in *P. antennata* remained to be identified, while GSTs were most likely to be completely found. For detoxification genes in three cerambycids derived from the transcriptomes, *P. antennata* had more relatives in each family, probably attributed to RNA–Seq of multiple tissues (*X. quadripes*: mixed female and male antennae (Ji et al., 2018); *R. horsfieldi*: six tissues (Zhao et al., 2020a); *P. antennata*: 12 tissues in this study). Furthermore, a wide tissue expression profile of most detoxification genes in *P. antennata* further supported the notion that the transcriptome of multiple tissues led to the identification of more detoxification genes. On the other hand, it was also possible that the breadth and/or differences of host plants among these beetles resulted in different sizes of detoxification genes. For example, *A. glabripennis* was a generalist with the larvae feeding on over 100 host plants (Meng et al., 2015).

Insect P450 is known as its role in insecticide resistance and thus is the most extensively studied detoxification gene family (Feyereisen, 1999; Dermauw et al., 2020). Among four P450 clans, both CYP3 and CYP4 commonly have species- or family-specific expansions, mainly contributing to the size differences of this gene family across insects. In contrast, Mito and CYP2 members are highly conserved among species (Feyereisen, 2006; Zhao et al., 2020b). Our phylogenetic analyses of P450s revealed a high degree of orthology between *P. antennata* and *A. glabripennis* (52 orthologous pairs), especially Mito and CYP2 clades associated with cuticle formation (Sztal et al., 2012), circadian rhythm (Sathyanarayanan et al., 2008), and the biosynthesis of juvenile hormones and 20-hydroxyecdysone (Feyereisen, 2011; Iga and Kataoka, 2012). However, no obvious clusters were found in the tree. These results suggest functional conservation of orthologous P450s between the two beetles.

In Coleoptera, members of CYP3 and CYP4 families mainly respond to insecticides and plant secondary metabolites. In three *Dendroctonus* bark beetles *D. valens*, *D. rhizophagus* and *D. armandi*, some CYP3 and CYP4 genes enriched in antennae and guts had inducible expression by host monoterpenes (Cano-Ramirez et al., 2013; Lopez et al., 2013; Dai et al., 2021). Similarly, *A. glabripennis* P450s expressed in larval guts were induced by feeding host plant sugar maple, as most of the genes belonged to CYP3 and CYP4 clans (McKenna et al., 2016). In the two CYP families, our study detected a number of genes expressed in antennae and abdomens by combinations of RNA–Seq and PCR approaches, possibly associated with the detoxification of host plant chemicals. In particular, some P450s specifically or highly expressed in the antennae of *P. antennata* may play important roles in the degradation of host volatiles. Focusing on *PantCYP4Q28* significantly enriched in male antennae, it was regarded as a candidate pheromone degrading enzyme (PDE), coupled with pheromone degrading functions of a male-antenna-specific gene *PdivCYP4AW1* in *Phyllopertha diversa* (Maibeche-Coisne et al., 2004). Outside the ability of P450s to metabolize plant chemicals, CYP3 and CYP4 enzymes that were broadly expressed in tissues have been demonstrated to have the involvement in insecticide resistance (Zhu et al., 2010; Zimmer et al., 2014). The majority of CYP3 and CYP4 genes in *P. antennata* also presented a wide tissue expression profile including abdomens with detoxification tissues, suggestive of their putative roles in the detoxification of insecticides.

In the COE gene family, the species-specific expansion mainly occurred in clades A, C and E (Zhao et al., 2020b). For example, 13 *A. glabripennis* COEs clustered into a group of clade A (coleopteran xenobiotic metabolizing enzymes), of which the majority of them were distributed on scaffold 498 with high conservation (69.40% mean amino acid identity) (McKenna et al., 2016). Six *P. antennata* COEs phylogenetically close to the *A. glabripennis* cluster formed a relatively small clade, and shared an average of 72.51% amino acid identity. Such the cluster and conservation were obtained in the two cerambycid beetles of clade E (β- and pheromone esterases). These findings suggest that cerambycid species possibly utilize gene duplications to generate a number of gene copies so as to adapt feeding host plants and the changeable habitats.

Previously, *PjapPDE* from *Popillia japonica* belonging to clade E was exclusive to male antennae and was capable of degrading a sex pheromone (R)-japonilure (Ishida and Leal, 2008). Focusing on 13 PantCOEs in this clade, we found that *PantCOE71* exhibited 8.36-fold higher expression in male antennae relative to females, suggesting its putative roles in inactivating female-specific compounds (e.g., sex pheromones). Except for clade E, esterases from other clades were involved in the degradation of odorant molecules, including clades D (integument esterases) and G (Lepidopteran juvenile hormone esterases) where some antenna-enriched esterases in Lepidoptera could degrade sex pheromones or plant odorants (He et al., 2014; He et al., 2015). In Coleoptera, clade F replaced clade G as candidate non-lepidopteran juvenile hormone esterases (Zhao et al., 2020b). In clades D and F, we sought to identify candidate odorant degrading enzymes (ODEs) in *P. antennata*. As shown in expression profiles, *PantCOE15*/*COE16*/*COE36* highly expressed in antennae were possibly associated with the removal of odorants and thus were identified as candidate ODEs. In addition, we noticed that most *PantCOEs* were enriched in thoraxes or abdomens, similar to the results in *X. quadripes* and *R. horsfieldi* (Zhao et al., 2020b). This could be explained by the fact that insect abdomen contains three important detoxification tissues (midgut, fat body and Malpighian tubules). Although thorax-enriched COEs were also found in other species (Zhao et al., 2020b), their putative functions in this tissue remain unknown and deserve to be further addressed.

As two major subclasses of Delta and Epsilon involved in the detoxification of xenobiotics including plant allelochemicals and insecticides, they accounted for a large proportion of GSTs in most insects (Zhao et al., 2020b; Gao et al., 2020). Meanwhile, due to the absence of Delta and Epsilon members in non-insect species (Sheehan et al., 2001), this further reflects their importance for survival and reproduction of insects. *P. antennata* possessed a similar proportion of GSTs in the two clades (62.50%) with the other three cerambycid beetles *A. glabripennis* (71.43%) (Mason et al., 2016), *R. horsfieldi* (65.00%) and *X. quadripes* (66.67%) (Zhao et al., 2020b), suggesting the requirements for detoxifying a variety of toxic compounds. The extensive expression of Delta and Epsilon genes in abdomens of *P. antennata* further conferred their resistance to xenobiotics, similar to the GST genes in *Tenebrio molitor* (Liu et al., 2015), *A. glabripennis* (Scully et al., 2013) and *X. quadripes* (Zhao et al., 2020b). Beyond the detoxification, insect GSTs have been also implied to have olfactory roles responsible for the degradation of odorants (Rogers et al., 1999). In *P. antennata*, the number (25 genes) of GSTs expressed in antennae was fewer compared to that in the antennal transcriptome of *D. melanogaster* (31 GSTs) (Younus et al., 2014), but more than those derived from antennal transcriptomes in *X. quadripes* (18 relatives) (Zhao et al., 2020b) and *Agrilus planipennis* (13 relatives) (Praveen et al., 2013). Hence, it is possible that antenna-enriched GSTs in *P. antennata* may be correlated with odorant degradation.

In conclusion, we sequenced the transcriptome of 12 tissues in *P. antennata*, generating 327,263,716 clean reads in males and 341,437,850 in females with a total of 100.28 G data. Transcriptome analysis combined with the bioinformatics-based search led to the yields of 215 detoxification genes, 107 of which were identified as DEGs. Phylogenetic analyses revealed that *P. antennata* detoxification genes shared high conservation with those in *A. glabripennis*, representing 52 P450, 31 COE and 11 GST orthologous pairs. Expression profiles identified a number of detoxification genes in abdomens, suggesting putative roles in xenobiotics metabolism. On the other hand, a certain number of detoxification genes were enriched in antennae, as important molecular candidates for odorant detection. Taken together, this study provides valuable resources for further investigating the adaptation of *P. antennata* to host plants and habitats, and identifies potential molecular targets involved in the detoxification and chemoreception.

## Data Availability

Raw data of the transcriptome presented in this study were deposited in the National Center for Biotechnology Information (NCBI) Sequence Read Archive (SRA) repository, with the BioProject accession number PRJNA821002. All nucleotide sequences of detoxification genes from *Pharsalia antennata* were deposited in the NCBI GenBank repository, with accession numbers OP314595–OP314700 for P450s, OP314701–OP314777 for COEs and OP314778–OP314809 for GSTs.
