# Supplementary material for: Identification and characterization of candidate detoxification genes in Pharsalia antennata Gahan (Coleoptera: Cerambycidae)
Source: Front Physiol. 2022 Sep 16;13:1015793. doi: 10.3389/fphys.2022.1015793 (PMC9523569; doi:10.3389/fphys.2022.1015793)
Supplement: Supplementary file 2 [file Table1.DOCX]

**Table S1**

| **Gene** | **Primer sequence (5'**–**3')** | |
| --- | --- | --- |
|  | **Forward primer** | **Reverse primer** |
| **RT–PCR analysis** | | |
| *CYP12T14* | ATGCAGCCTAGAACAGCACAAC | GTTGGTGCTCTGGTACGTCATC |
| *CYP12T15* | AAGTATGGATAAAGTCGCCGGT | CCAACGTTTTTCCTGTCGTATC |
| *CYP12T16* | ATCGCCATATCTTAAGGCAGC | AAGTGGATCCATGGTTCCGTAC |
| *CYP302A1* | GTTGTGAAGGAAAACATCCCCT | CAAGAATTGGTCTCCAAAGCAG |
| *CYP334H5* | AATCAAGCCAATAATACGGCTG | AACTGCATTTGCATGTTCTGGT |
| *CYP15A1* | GATGCTCCTCTTGCTCTGCAT | GCGGGTCTCTTCCTGTATTTTC |
| *CYP18A1* | CAGTTCTGGTAATAGTTCGCGG | GCCTTGAGGACGTAGAGGAACT |
| *CYP305A1* | AGTTACATACTGTTAGCGGCCG | CAAACTGCGGAGATGAGTCACT |
| *CYP306A1* | CGTGGGACAGAGATGAGCATAT | AACGGTAGGGTTTTTGGCTAAG |
| *CYP307B1* | CGGTCACCTTAACATCATCGAT | GTGTCGGACCAATCACAGAAAG |
| *CYP345M4* | AACTCCACGGACCAACCCTAT | AAGACAAAGAGCAGTCCCGCT |
| *CYP345N1* | AAGAAAAGGAATGTGCACTACGAG | GCACATTTCGCTATAACGTCTGT |
| *CYP347H1* | AGCACACGTTCGTAATGAAAGG | GTTGTTGGTCAATGCCATCATT |
| *CYP347H5* | GGTACTGGAAAAGTAGGGGCAT | AAGGGCTGTATCTTCTTGCCTT |
| *CYP6BJ9* | CTTGGGAGTGATAACCGCACT | CAAGGATGACCCCACAGTCAG |
| *CYP6BJ14* | CATGACCAAGGACTTCCACAAC | GGCAGCTACTTCAGGGAAGTTC |
| *CYP6BJ16* | CTACTTCGGATCCTTTGTGCTG | GGTTCCTCCACTTAACACCACC |
| *CYP6BJ17* | GTCATCGGTTCCTGTGCATTC | AACAGAGCGAAGGTCATGGTG |
| *CYP6BJ49* | ATGGTGCTTGACTTGGATATGG | AGAAGGCCTCTTTCATCAGCTC |
| *CYP6BJ61* | CAATACCCTTCGGAAACTTGG | ACCGCAAGAGCCTATCATGTC |
| *CYP6BJ63* | CTTAGTCGACCTTGTGGGTTTG | GCTGCTCAAGATATTCAGCCAT |
| *CYP6BJ64* | AGAGAAAAAATGCGCCCTATCT | ACCGCTTCCTTTTTTTCAAGTG |
| *CYP6BJ-F3* | ACTCAGGGATGAAATCAAAGAGG | GCCGACTTTGACCTGCATTAT |
| *CYP6BJ66* | CGGCTTGGAATGTAACAGCTT | ATGTCTTGATGTTGGGCCAAC |
| *CYP6BJ67* | AGCCTCTTTATGGAGGTCTTGG | ACAGTCTGCCAATGTCTGGAAC |
| *CYP6BQ32* | GACTTGATCCTCGGAAGGAAAC | AGATCGAGTCGGGGTTCTTTAG |
| *CYP6EF8* | CACTACCACAACGAAAAGGACG | AACTTCGATACGTTGTCGTTGG |
| *CYP6HD1* | AGATTATCAAGCGCGTGCTAAT | AGGTCTCGAATTCTTTTGGCAT |
| *CYP6HD2* | CTCGAGCCAGTGATACCTTTTG | GGAGTGCCATCAGTAGCGTAAG |
| *CYP6TW8* | AACATGGCCAAAAGAGGAACTT | CTTAATTCCAAAAGCAGCGGAT |
| *CYP6TW-F1* | GCTATGAATGAAATGACTTATCTGG | TCAAACTCTAGTCACATTTACCCAC |
| *CYP9Z47* | GATACTTGGAACGATAGTCGCG | GAAATGCTGAACGAAGTTCTCG |
| *CYP9Z198* | AGCAGCAAGATGAGAAGCATGT | AGGAATTCCTTTCCTCGCTTC |
| *CYP9Z-F5* | GAGGGCCATGTTTGTCTTAATG | AGGTTTTCCCAAGTCACATTCC |
| *CYP349E3* | CTTTAGTGCTGGTGGTGACAGC | GCGTAGGTCTTCAGTACGTTCG |
| *CYP349F15* | CACTTCGTTGGAAGCCCATAT | CAATCCACTTGGACACAGGCT |
| *CYP352A1* | ACATGCCACCAGATTTGCTAAT | GTGAGCATGTGGTCCTTGAATT |
| *CYP4AA13* | AACATACCTGGTCCTCCAGCTT | AACGCCTAATATTGCCCTGTG |
| *CYP4BN65* | GCCTATAATCGGATGTGCTTTG | GGCTGCTTCACATATAACGTCC |
| *CYP4EJ1* | CAGATTCTGAAGCCTTGCAAGT | CAAATCTTTTGTAGGGCGACTG |
| *CYP4G104* | CGTCAGGAGCAGTTTTATCTGC | GTGCAATCAGTTTGCGATGTG |
| *CYP4G105* | AGAATATGGTTGGGTCCCAAAC | CCACATCGACGTGAACTTGAAT |
| *CYP4NQ6* | GAATCCACTTGAGTTATTGCCG | AGCAAAAACCCTCTCCATGACT |
| *CYP4NQ22* | CGACAAAAGATTTTCCACAAGG | ATCCACCGTAGCTTTTCCTTCT |
| *CYP4Q28* | GGAAATTCCAGGACCAAAATCT | AGGTCGATCGCAATTTTTCTCT |
| *CYP4Q81* | ACGGGAATGGATCAGATTTTTC | AAGGCTGGTGACACATTTTCCT |
| *CYP4Q82* | GTTATTGGGCTGATAGCACTGC | CGTTCTCAAGCACCTTTACCAG |
| *CYP4Q83* | ATGCTTTGCTCTGATGGCACT | CCACATAGGGCAGCCTTTATC |
| *COE4* | ATACCAGCTGATGTTCCCCCT | CATGTCCCACAGGGCTACATT |
| *COE5* | CGGTGATGCACTTGGAATTAAG | GAAAGCCCAAAGCACCTAATCT |
| *COE8* | CCACAACCTACCACAGATCCTG | AGGAATGGGGAAGTTGCCTAG |
| *COE9* | CAACAGGATCGAACAAGCCTAG | CAAGGCCTCTCTATCAGTGCTG |
| *COE12* | GCGACACCATCTGTTTAAGTCC | CAAGGACGATGTCTTTGTCCAT |
| *COE13* | GAGGGTCAAATAAAGGGACACG | CCTGGTATAACGCTATCTCCGG |
| *COE15* | CGCCATAATAATATGCCTCAGC | CACCGAAGTCGCACTGTAGAAT |
| *COE19* | CTGACAGGCTATTTGTTGCCTC | GCTCCAAAAGTCAATAGGGGTC |
| *COE21* | GTTGCAGTGGGTTCAAGAAAAC | GGCTTCCTCTGAATTTATGCCT |
| *COE24* | CCAGTTGGCAAGTTGAGACTTC | CGTTGTCCTGTACCCATTTCAG |
| *COE26* | GTAACATTCACGCCAGAAGCC | GAAAGCCGAAAATTCCCAGTC |
| *COE27* | GGAAAAATACTGGGCAGCAAG | CAAACCCCAGTTACCAGATGC |
| *COE28* | CTCAGCATTGGCTCAGGAAGT | CCATGTTTCCAGGTAGAACCG |
| *COE29* | ACACCACGTTTTACGCTTTCC | AGCGTTGATCTTTCAAGCCAT |
| *COE30* | CAGATGATCCCGTCGTCAGTT | CAAATGGACCCACTCGGTAAT |
| *COE31* | CAGTACCACTTGCAGAAGAGCC | GTAGACAGGAACCCGAAAGGTC |
| *COE32* | ATTGCTTCTGCAGCTGAATCAG | CTGCAGTCAGAAAACCAAAAGG |
| *COE34* | CGTTCTTCAGGTTCTCTGGGAT | GATCACCTCCGAAACTGTGGAT |
| *COE35* | GTGATCGAATATACGCTGCCTT | ATCTTTCATTCCGTTGTTGCC |
| *COE36* | GGAACTGGGAGAATCTCTACCG | GTTTTGCTGCTCTGTTCACTGC |
| *COE37* | GCTAAACCGCCTTTAGGAGATC | GGTAAGTAACTGACGCAGCACC |
| *COE39* | CACCACCAGTAGGCGAATTAAG | AGCACTTTCTCCCGAAAGTGTT |
| *COE40* | CGATTGGGAGCTCTAGGATTTC | CAGCATCAGGAGTAATTGCGTT |
| *COE41* | CTAAGGTTTCAACCACCAGTTCC | CTCCGCCAAACAGGTTGATAT |
| *COE42* | CAAGCCAAGATGATCCGCTAC | CATCTTCGGTGCTAAGGAACC |
| *COE44* | AGCATTGGCTCAGGATGTCAC | AATGCCAGGTTCTGGTCCTTC |
| *COE45* | GTGGGTTTAGAGACGGAGCTTC | CGGTCATAGTTCACCGTTTGTG |
| *COE47* | AGACATACCGTACGCAGCTCC | GATCCACTGAAGGGCAAGATG |
| *COE49* | AACGAGGATGATCTGCCAGAG | GTTGGGGGTTACCAACACAAC |
| *COE51* | AGCGCTTACCACCATCAATAAC | GGATCACCACCGAAATATTCG |
| *COE52* | GAGGACTGTTTGTACCTGAGCG | CGAGCATAGTCTTGACGAGTCC |
| *COE53* | ACAATGAAGCGCCAGTTGTAAC | CCAGGAATCACATCATCCTCTG |
| *COE55* | GCCATGCTTTACAGTTTGTTCC | GCTAAGGAAACCAAACGAAGCT |
| *COE56* | ACTGCATTTGAAGGGATTCCTT | ACGATTGATCCTTCAACCCATT |
| *COE64* | ATGGCAGCTCCAGTTGTACTTG | CGATAACACCAGCCACTTTCTG |
| *COE65* | ATACCGGGGAATTTGGGTCT | CCTCAATTATGGGCAGCCAT |
| *COE67* | TGATCAAGAATTTACCACGCC | GTGGTGGTATAGCGTATACATCG |
| *COE68* | CCAGACGTTCCAGAACACAAAG | CAAGGGGTGGCAATATTTTCTC |
| *GSTd1* | GGTTCGTTTAGCGGCTAAAAC | AGCTAAAGTGAGATGGTCGCCT |
| *GSTd3* | AATAGAATTGAACCCCAAGCCT | ATAACATGCAGCTTCACAAGCC |
| *GSTd4* | GAACATCTTACGCCCGAATTC | AGCTGTTGCCTTTACTTTGGC |
| *GSTd5* | CCTGCAGGGCAGTCTTTTTAG | CGGCTAAAGTAAGATGGTCGC |
| *GSTd7* | GCGTTGTCCCTTTAAACAACAG | AAGCTGACCTCCAAAAAAGGTC |
| *GSTd11* | AATATGGCAAAGACGACAGCC | AGCACACGATTTCTTCCGAAG |
| *GSTe2* | ATGGCGCCTAAATTGTACTACG | GCATACCCTTGTGTGAGCAGAT |
| *GSTe5* | ATGTCATCAACGCATACCTGGT | ATCAAAGCGGCAAACTGATCT |
| *GSTe7* | AAGTCCACCAGTAAGGGCATCT | AGCGACGTAGGTGCTTCTTTCT |
| *GSTe8* | CGAATAGAGGTGAACACCATGC | CGGTAACTTTAGGGAACCGACT |
| *GSTo1* | GGTTCTAAAGAGCCTCCCAGAG | GTGTGCCTCTCTGAGCTAATGC |
| *GSTo2* | ATTTTGTCCATATGCCCAACG | CCTTTCACACCAAGGCCATAT |
| *GSTs1* | ATTTCGAACTCACAGGCCTTG | CAAGTTATTCGGCCACCAACT |
| *GSTs5* | GCCTGCTTTGAAACCATCTATG | AGTTCTCGGTCGTTTATCCACC |
| *GSTz1* | AGGTTCCCACGTTGTCGATAG | CTGATGATCGGGATGAGCTTC |
| *RPS3* | GTCCGAAGATGGATACTCTGGTG | CAAAGGTTTCTTCGGTCCAGTC |
| **qPCR analysis** | | |
| *COE5* | TTGGTCAGGATACAGAGTTC | CCATCTTCAGTATTGAGTCC |
| *COE15* | GCTGATGTGTACTCTGAC | GAAGGTAGAAGTGACTGTAG |
| *COE27* | CTGTAGAATTAGCGGAATGG | CATCTGCGAACGGTAATC |
| *COE36* | GATAAGCCTGTGGACAAATC | AAGGTAGAAGTGACTGTAGC |
| *COE55* | GCTATTAAGAACAGCCTTGG | CCGCAGAATTAAACCCTATC |
| *GSTd3* | GCGACACCCTTACTATTG | CATATCCTGGAGCAGTATC |
| *GSTd4* | GCTTCCTGACTGATTCTG | CTTCTTCGTAACCTGGAG |
| *GSTd7* | AGTGCTCCATCTAGGTCAG | GGCTGTCATATACTACGAATCC |
| *GSTe2* | TCGCTTCCAACAGATTCC | GCTTGCTCTTAACCATTCC |
| *GSTe8* | TAACCAGCGACTCTACTTC | TCTTCTCCAGCAGCATAG |
| *GSTs1* | GGCAGGACCTCTATTTAATG | CGTATTCCAACATAGACACC |
| *CYP18A1* | CTTATTCGAGGGCAAAGATC | CTCTCATTGTGTCTGGATTG |
| *CYP345N1* | GATGGCACTACATTACGATG | TACCTTGACTCCTAGCATTC |
| *CYP347H1* | ACTGAACCCTACACTATGAC | CTCTGGAATAAACTTGTCGG |
| *CYP6BJ61* | CAGTACATTGGCATGAAGAG | TCTCAGAATACAGACCAGAC |
| *CYP6BJ64* | GGAGATACGGTTACCTACAG | GAAGAAGACGAAGCATTGAG |
| *CYP6BJ66* | ACTACAAGATACCTGGTGAG | GTAAGTGTGTCTTCCCTTTC |
| *CYP6EF8* | ATATGGGCAGGGAGATATTC | GTACGGCATCATAGAAGAAC |
| *CYP349E3* | CATTCCTGGATTTGCTCTTG | CTATGACTTCCTCGTAGACC |
| *CYP349F15* | GAAACTACCAGGACCATTTG | CCAGACCCGAAATATACTTG |
| *CYP4AA13* | AACCCAGTGTAGTTACTCTG | ATAATGAGGCAGTCTGTGAG |
| *CYP4EJ1* | TTATGTACCACCTACGAGAC | CCATTTATCCACTCCACTTG |
| *CYP4NQ6* | CTGGATGTAATATGTGAGGC | GGATACAGTGAATGGTGAAC |
| *CYP4Q28* | GTTCTCAACGACGATTGTAC | TCAACTTCTTCCCTGATACC |
| *CYP4Q83* | CAAACAGCACGAGAACAG | AATCCAGCATAGCCAGAC |
| *RPS3* | GGCTTGTTATGGTGTCTTG | TTCATGGACTTCGCTCTC |
| *RPL10* | GTCGTGCCAAATTCAAGT | AACATTACAACCGTCGTG |
