# Supplementary material for: Identification and characterization of candidate detoxification genes in Pharsalia antennata Gahan (Coleoptera: Cerambycidae)
Source: Front Physiol. 2022 Sep 16;13:1015793. doi: 10.3389/fphys.2022.1015793 (PMC9523569; doi:10.3389/fphys.2022.1015793)
Supplement: Supplementary file 3 [file Table2.DOCX]

**Table S2**

| **Gene** | **Accession number** | **ORF (AA)** | **FL** | **NCBI Blast Hit (Reference/Name/Species)** | ***E*-value** | **Identity (%)** |
| --- | --- | --- | --- | --- | --- | --- |
| **Cytochrome P450 (CYP)** | | | | | | |
| CYP12T14 | OP314595 | 539 | Yes | XP_018572395.1 cytochrome P450 CYP12A2 [*Anoplophora glabripennis*] | 0.0 | 74.40 |
| CYP12T15 | OP314596 | 539 | Yes | XP_018567976.1 probable cytochrome P450 12a4, mitochondrial [*Anoplophora glabripennis*] | 0.0 | 74.81 |
| CYP12T16 | OP314597 | 168 | No | XP_018567976.1 probable cytochrome P450 12a4, mitochondrial [*Anoplophora glabripennis*] | 4e-89 | 78.11 |
| CYP301A1 | OP314598 | 520 | Yes | XP_018568722.1 probable cytochrome P450 301a1, mitochondrial [*Anoplophora glabripennis*] | 0.0 | 91.52 |
| CYP301B1 | OP314599 | 516 | Yes | XP_018568698.1 probable cytochrome P450 49a1 [*Anoplophora glabripennis*] | 0.0 | 88.95 |
| CYP302A1 | OP314600 | 506 | Yes | XP_018574126.1 cytochrome P450 302a1, mitochondrial-like [*Anoplophora glabripennis*] | 0.0 | 87.75 |
| CYP314A1 | OP314601 | 490 | Yes | XP_023021215.1 ecdysone 20-monooxygenase isoform X2 [*Leptinotarsa decemlineata*] | 0.0 | 65.17 |
| CYP315A1 | OP314602 | 469 | Yes | XP_018578926.1 cytochrome P450 315a1, mitochondrial [*Anoplophora glabripennis*] | 0.0 | 90.19 |
| CYP334H5 | OP314603 | 593 | Yes | XP_023019969.1 probable cytochrome P450 301a1, mitochondrial [*Leptinotarsa decemlineata*] | 0.0 | 62.06 |
| CYP353A1 | OP314604 | 473 | Yes | XP_018577263.1 probable cytochrome P450 49a1 [*Anoplophora glabripennis*] | 0.0 | 84.89 |
| CYP49A1 | OP314605 | 551 | Yes | XP_018570997.1 probable cytochrome P450 49a1 [*Anoplophora glabripennis*] | 0.0 | 89.47 |
| CYP15A1 | OP314606 | 492 | Yes | NP_001308597.1 cytochrome P450 15A1 precursor [*Tribolium castaneum*] | 0.0 | 72.36 |
| CYP18A1 | OP314607 | 524 | Yes | XP_018564416.1 cytochrome P450 18a1 [*Anoplophora glabripennis*] | 0.0 | 97.33 |
| CYP305A1 | OP314608 | 492 | Yes | XP_019871644.1 PREDICTED: LOW QUALITY PROTEIN: probable cytochrome P450 305a1 [*Aethina* *tumida*] | 0.0 | 67.14 |
| CYP306A1 | OP314609 | 499 | Yes | XP_018564384.1 cytochrome P450 306a1 [*Anoplophora glabripennis*] | 0.0 | 83.47 |
| CYP307A2 | OP314610 | 210 | No | XP_018568738.1 cytochrome P450 307a1-like [*Anoplophora glabripennis*] | 2e-145 | 95.69 |
| CYP307B1 | OP314611 | 427 | Yes | XP_018568107.1 cytochrome P450 307a1-like [*Anoplophora glabripennis*] | 0.0 | 73.35 |
| CYP345F4 | OP314612 | 499 | Yes | XP_018562134.1 cytochrome P450 6k1 isoform X2 [*Anoplophora glabripennis*] | 0.0 | 88.78 |
| CYP345M2 | OP314613 | 502 | Yes | XP_018561047.1 cytochrome P450 6k1 [*Anoplophora glabripennis*] | 0.0 | 69.42 |
| CYP345M3 | OP314614 | 461 | No | XP_018561047.1 cytochrome P450 6k1 [*Anoplophora glabripennis*] | 0.0 | 77.22 |
| CYP345M4 | OP314615 | 501 | Yes | XP_018561047.1 cytochrome P450 6k1 [*Anoplophora glabripennis*] | 0.0 | 64.52 |
| CYP345M5 | OP314616 | 502 | Yes | XP_018561047.1 cytochrome P450 6k1 [*Anoplophora glabripennis*] | 0.0 | 77.67 |
| CYP345N1 | OP314617 | 503 | Yes | QYA71972.1 cytochrome P450 [*Anoplophora glabripennis*] | 0.0 | 86.08 |
| CYP345N7 | OP314618 | 496 | Yes | QYA71972.1 cytochrome P450 [*Anoplophora glabripennis*] | 0.0 | 67.33 |
| CYP347H1 | OP314619 | 490 | Yes | QYA71971.1 cytochrome P450 [*Anoplophora glabripennis*] | 0.0 | 84.91 |
| CYP347H4 | OP314620 | 486 | Yes | XP_018565210.1 cytochrome P450 6j1 [*Anoplophora glabripennis*] | 0.0 | 82.30 |
| CYP347H5 | OP314621 | 487 | Yes | XP_018565210.1 cytochrome P450 6j1 [*Anoplophora glabripennis*] | 0.0 | 72.43 |
| CYP347H6 | OP314622 | 591 | Yes | QYA71971.1 cytochrome P450 [*Anoplophora glabripennis*] | 0.0 | 70.26 |
| CYP347H7 | OP314623 | 415 | No | QYA71971.1 cytochrome P450 [*Anoplophora glabripennis*] | 0.0 | 74.46 |
| CYP413B1 | OP314624 | 469 | Yes | XP_018579609.1 cytochrome P450 6B4 [*Anoplophora glabripennis*] | 0.0 | 72.59 |
| CYP6BH3 | OP314625 | 502 | Yes | XP_018569720.1 probable cytochrome P450 6a13 [*Anoplophora glabripennis*] | 0.0 | 79.12 |
| CYP6BH9 | OP314626 | 502 | Yes | XP_018574914.2 cytochrome P450 6A1-like [*Anoplophora glabripennis*] | 0.0 | 68.19 |
| CYP6BH22 | OP314627 | 521 | Yes | XP_018574914.2 cytochrome P450 6A1-like [*Anoplophora glabripennis*] | 0.0 | 70.36 |
| CYP6BJ9 | OP314628 | 512 | Yes | XP_018576967.1 cytochrome P450 6a9 [*Anoplophora glabripennis*] | 0.0 | 83.59 |
| CYP6BJ14 | OP314629 | 512 | Yes | XP_023311070.1 probable cytochrome P450 6a14 [*Anoplophora glabripennis*] | 0.0 | 84.57 |
| CYP6BJ16 | OP314630 | 508 | Yes | XP_018576930.1 probable cytochrome P450 6a14 [*Anoplophora glabripennis*] | 0.0 | 74.50 |
| CYP6BJ17 | OP314631 | 322 | No | XP_018576919.1 probable cytochrome P450 6a14 isoform X2 [*Anoplophora glabripennis*] | 0.0 | 82.97 |
| CYP6BJ48 | OP314632 | 322 | No | QYA71986.1 cytochrome P450 [*Anoplophora glabripennis*] | 0.0 | 75.47 |
| CYP6BJ49 | OP314633 | 445 | No | XP_023311082.1 probable cytochrome P450 6a13 [*Anoplophora glabripennis*] | 0.0 | 80.22 |
| CYP6BJ52 | OP314634 | 378 | No | XP_018576754.1 cytochrome P450 6a2 isoform X1 [*Anoplophora glabripennis*] | 0.0 | 76.46 |
| CYP6BJ54 | OP314635 | 361 | No | XP_023310272.1 probable cytochrome P450 6a13 [*Anoplophora glabripennis*] | 0.0 | 84.21 |
| CYP6BJ55-F1 | OP314636 | 182 | No | XP_018576916.1 probable cytochrome P450 6a14 isoform X1 [*Anoplophora glabripennis*] | 5e-98 | 76.80 |
| CYP6BJ61 | OP314637 | 506 | Yes | XP_023311076.1 LOW QUALITY PROTEIN: probable cytochrome P450 6a14 [*Anoplophora glabripennis*] | 0.0 | 66.80 |
| CYP6BJ62 | OP314638 | 513 | Yes | XP_018576939.1 probable cytochrome P450 6a14 [*Anoplophora glabripennis*] | 0.0 | 70.76 |
| CYP6BJ63 | OP314639 | 513 | Yes | XP_018576632.1 cytochrome P450 6a2-like [*Anoplophora glabripennis*] | 0.0 | 69.84 |
| CYP6BJ64 | OP314640 | 512 | Yes | QYA71976.1 cytochrome P450 [*Anoplophora glabripennis*] | 0.0 | 82.03 |
| CYP6BJ65 | OP314641 | 510 | Yes | QYA71986.1 cytochrome P450 [*Anoplophora glabripennis*] | 0.0 | 75.73 |
| CYP6BJ66 | OP314642 | 351 | No | XP_018576951.1 probable cytochrome P450 6a13 [*Anoplophora glabripennis*] | 0.0 | 75.78 |
| CYP6BJ67 | OP314643 | 482 | No | XP_018576967.1 cytochrome P450 6a9 [*Anoplophora glabripennis*] | 0.0 | 83.01 |
| CYP6BJ-F1 | OP314644 | 142 | No | XP_018577090.1 probable cytochrome P450 6a14 isoform X1 [*Anoplophora glabripennis*] | 9e-60 | 62.68 |
| CYP6BJ-F2 | OP314645 | 247 | No | XP_023311070.1 probable cytochrome P450 6a14 [*Anoplophora glabripennis*] | 3e-137 | 75.71 |
| CYP6BJ-F3 | OP314646 | 136 | No | XP_018576916.1 probable cytochrome P450 6a14 isoform X1 [*Anoplophora glabripennis*] | 3e-76 | 78.68 |
| CYP6BJ-F4 | OP314647 | 173 | No | XP_018576916.1 probable cytochrome P450 6a14 isoform X1 [*Anoplophora glabripennis*] | 8e-88 | 72.25 |
| CYP6BQ32 | OP314648 | 506 | Yes | XP_018573006.1 probable cytochrome P450 6a13 [*Anoplophora glabripennis*] | 0.0 | 81.84 |
| CYP6BS4 | OP314649 | 357 | No | XP_018571884.1 cytochrome P450 6a9-like [*Anoplophora glabripennis*] | 0.0 | 92.00 |
| CYP6EF8 | OP314650 | 505 | Yes | XP_023310525.1 cytochrome P450 6a2-like isoform X1 [*Anoplophora glabripennis*] | 0.0 | 90.69 |
| CYP6HD1 | OP314651 | 516 | Yes | QYA71966.1 cytochrome P450 [*Anoplophora glabripennis*] | 0.0 | 92.64 |
| CYP6HD2 | OP314652 | 512 | Yes | QYA71977.1 cytochrome P450 [*Anoplophora glabripennis*] | 0.0 | 82.23 |
| CYP6TW5 | OP314653 | 507 | Yes | XP_018571707.1 probable cytochrome P450 6a13 [*Anoplophora glabripennis*] | 0.0 | 79.68 |
| CYP6TW8 | OP314654 | 449 | No | XP_018571701.1 probable cytochrome P450 6a23 [*Anoplophora glabripennis*] | 0.0 | 83.78 |
| CYP6TW9 | OP314655 | 372 | No | XP_018571700.1 probable cytochrome P450 6a20 [*Anoplophora glabripennis*] | 0.0 | 86.02 |
| CYP6TW-F1 | OP314656 | 171 | No | XP_018571701.1 probable cytochrome P450 6a23 [*Anoplophora glabripennis*] | 1e-104 | 85.96 |
| CYP6TW-F2 | OP314657 | 138 | No | XP_018571701.1 probable cytochrome P450 6a23 [*Anoplophora glabripennis*] | 1e-76 | 81.16 |
| CYP9BP1 | OP314658 | 468 | No | NP_001352311.1 cytochrome P450 9e2 [*Anoplophora glabripennis*] | 0.0 | 59.00 |
| CYP9BQ1 | OP314659 | 542 | No | XP_018573794.1 cytochrome P450 9e2 [*Anoplophora glabripennis*] | 0.0 | 76.68 |
| CYP9D15 | OP314660 | 533 | Yes | XP_019870850.1 PREDICTED: cytochrome P450 9e2-like [*Aethina tumida*] | 2e-128 | 38.40 |
| CYP9HE1-P | OP314661 | 280 | No | XP_019875691.1 PREDICTED: cytochrome P450 9e2-like isoform X2 [*Aethina tumida*] | 1e-96 | 51.25 |
| CYP9Y13 | OP314662 | 523 | Yes | XP_018571685.1 cytochrome P450 9e2 isoform X2 [*Anoplophora glabripennis*] | 0.0 | 74.44 |
| CYP9Z47 | OP314663 | 528 | Yes | XP_018570837.1 cytochrome P450 9e2-like [*Anoplophora glabripennis*] | 0.0 | 74.00 |
| CYP9Z198 | OP314664 | 361 | No | XP_018570837.1 cytochrome P450 9e2-like [*Anoplophora glabripennis*] | 0.0 | 73.06 |
| CYP9Z199 | OP314665 | 534 | Yes | XP_023310975.1 cytochrome P450 9e2 [*Anoplophora glabripennis*] | 0.0 | 74.86 |
| CYP9Z200 | OP314666 | 467 | No | XP_018561888.1 cytochrome P450 9e2-like [*Anoplophora glabripennis*] | 0.0 | 75.16 |
| CYP9Z-F1 | OP314667 | 338 | No | XP_018570837.1 cytochrome P450 9e2-like [*Anoplophora glabripennis*] | 1e-145 | 62.13 |
| CYP9Z-F2 | OP314668 | 317 | No | XP_018570837.1 cytochrome P450 9e2-like [*Anoplophora glabripennis*] | 3e-150 | 66.25 |
| CYP9Z-F3 | OP314669 | 257 | No | XP_018570837.1 cytochrome P450 9e2-like [*Anoplophora glabripennis*] | 2e-117 | 67.32 |
| CYP9Z-F4 | OP314670 | 208 | No | XP_023310975.1 cytochrome P450 9e2 [*Anoplophora glabripennis*] | 2e-110 | 75.00 |
| CYP9Z-F5 | OP314671 | 191 | No | XP_018570837.1 cytochrome P450 9e2-like [*Anoplophora glabripennis*] | 2e-77 | 63.87 |
| CYP9Z-F6 | OP314672 | 172 | No | XP_023310975.1 cytochrome P450 9e2 [*Anoplophora glabripennis*] | 1e-91 | 75.58 |
| CYP9Z-F7 | OP314673 | 148 | No | XP_018570839.1 cytochrome P450 9e2 [*Anoplophora glabripennis*] | 4e-56 | 63.51 |
| CYP9Z-F8 | OP314674 | 103 | No | XP_019875691.1 PREDICTED: cytochrome P450 9e2-like isoform X2 [*Aethina tumida*] | 1e-52 | 72.82 |
| CYP9Z-F9 | OP314675 | 99 | No | XP_023312541.1 cytochrome P450 9e2-like, partial [*Anoplophora glabripennis*] | 5e-49 | 80.41 |
| CYP3016C1 | OP314676 | 355 | No | XP_018563755.1 cytochrome P450 4C1-like [*Anoplophora glabripennis*] | 0.0 | 81.97 |
| CYP3016C4 | OP314677 | 498 | Yes | XP_018563755.1 cytochrome P450 4C1-like [*Anoplophora glabripennis*] | 0.0 | 76.41 |
| CYP3016C-F1 | OP314678 | 271 | No | XP_018563755.1 cytochrome P450 4C1-like [*Anoplophora glabripennis*] | 4e-100 | 56.81 |
| CYP349E3 | OP314679 | 502 | Yes | QYA71967.1 cytochrome P450 [*Anoplophora glabripennis*] | 0.0 | 79.28 |
| CYP349F1 | OP314680 | 357 | No | XP_018565098.2 cytochrome P450 4C1-like [*Anoplophora glabripennis*] | 0.0 | 75.91 |
| CYP349F15 | OP314681 | 507 | No | XP_018565098.2 cytochrome P450 4C1-like [*Anoplophora glabripennis*] | 0.0 | 67.79 |
| CYP352A1 | OP314682 | 501 | Yes | NP_001352313.1 cytochrome P450 4c3-like [*Anoplophora glabripennis*] | 0.0 | 84.57 |
| CYP411A1 | OP314683 | 412 | No | NP_001352375.1 cytochrome P450 4c3-like [*Anoplophora glabripennis*] | 0.0 | 86.65 |
| CYP434B1 | OP314684 | 418 | No | XP_023022449.1 cytochrome P450 4V2-like [*Leptinotarsa decemlineata*] | 9e-150 | 50.61 |
| CYP4AA13 | OP314685 | 471 | Yes | XP_018572296.1 probable cytochrome P450 4aa1 [*Anoplophora glabripennis*] | 0.0 | 83.01 |
| CYP4BN61-F | OP314686 | 346 | No | XP_023309749.1 cytochrome P450 4c3-like isoform X1 [*Anoplophora glabripennis*] | 0.0 | 84.10 |
| CYP4BN65 | OP314687 | 501 | Yes | XP_023309751.1 cytochrome P450 4d2 [*Anoplophora glabripennis*] | 0.0 | 76.25 |
| CYP4BN66 | OP314688 | 502 | Yes | QYA71980.1 cytochrome P450 [*Anoplophora glabripennis*] | 0.0 | 79.04 |
| CYP4BR6 | OP314689 | 501 | Yes | XP_023310211.1 cytochrome P450 4d2-like [*Anoplophora glabripennis*] | 0.0 | 90.62 |
| CYP4EJ1 | OP314690 | 490 | Yes | QYA71991.1 cytochrome P450 [*Anoplophora glabripennis*] | 0.0 | 81.22 |
| CYP4G104 | OP314691 | 562 | Yes | NP_001352320.1 cytochrome P450 4g15-like [*Anoplophora glabripennis*] | 0.0 | 94.84 |
| CYP4G105 | OP314692 | 536 | Yes | XP_018572314.1 cytochrome P450 4g15-like [*Anoplophora glabripennis*] | 0.0 | 87.64 |
| CYP4NQ6 | OP314693 | 492 | Yes | XP_023309752.1 cytochrome P450 4V2-like isoform X1 [*Anoplophora glabripennis*] | 0.0 | 80.74 |
| CYP4NQ22 | OP314694 | 494 | Yes | XP_023309752.1 cytochrome P450 4V2-like isoform X1 [*Anoplophora glabripennis*] | 0.0 | 67.21 |
| CYP4Q26 | OP314695 | 491 | Yes | XP_018561986.1 cytochrome P450 4C1-like isoform X2 [*Anoplophora glabripennis*] | 0.0 | 75.66 |
| CYP4Q28 | OP314696 | 491 | Yes | QYA71958.1 cytochrome P450 [*Anoplophora glabripennis*] | 0.0 | 79.84 |
| CYP4Q81 | OP314697 | 506 | Yes | QYA71981.1 cytochrome P450 [*Anoplophora glabripennis]* | 0.0 | 81.78 |
| CYP4Q82 | OP314698 | 491 | Yes | XP_023309989.1 cytochrome P450 4C1-like [*Anoplophora glabripennis*] | 0.0 | 73.06 |
| CYP4Q83 | OP314699 | 491 | Yes | XP_023309989.1 cytochrome P450 4C1-like [*Anoplophora glabripennis*] | 0.0 | 87.37 |
| CYP4Q-F | OP314700 | 157 | No | XP_023309989.1 cytochrome P450 4C1-like [*Anoplophora glabripennis*] | 4e-72 | 70.70 |
| **Carboxylesterase (COE)** | | | | | | |
| COE1 | OP314701 | 124 | No | XP_018563677.1 cholinesterase 1 [*Anoplophora glabripennis*] | 3e-60 | 81.45 |
| COE2 | OP314702 | 108 | No | XP_023020261.1 neuroligin-4, X-linked-like, partial [*Leptinotarsa decemlineata*] | 7e-73 | 100.00 |
| COE3 | OP314703 | 740 | Yes | XP_018571655.1 neurotactin [*Anoplophora glabripennis*] | 0.0 | 91.76 |
| COE4 | OP314704 | 672 | Yes | XP_018561763.1 liver carboxylesterase 1 [*Anoplophora glabripennis*] | 0.0 | 85.88 |
| COE5 | OP314705 | 581 | Yes | QYA71947.1 carboxylesterase [*Anoplophora glabripennis*] | 0.0 | 88.98 |
| COE6 | OP314706 | 263 | No | XP_018562621.1 venom carboxylesterase-6-like [*Anoplophora glabripennis*] | 3e-143 | 80.08 |
| COE7 | OP314707 | 1292 | Yes | XP_008192968.1 PREDICTED: neuroligin-3 [*Tribolium castaneum*] | 0.0 | 55.32 |
| COE8 | OP314708 | 569 | Yes | XP_023312469.1 esterase E4-like [*Anoplophora glabripennis*] | 0.0 | 89.28 |
| COE9 | OP314709 | 534 | Yes | XP_018575116.1 esterase B1 [*Anoplophora glabripennis*] | 0.0 | 72.66 |
| COE10 | OP314710 | 854 | Yes | XP_018570116.1 neuroligin-4, Y-linked isoform X1 [*Anoplophora glabripennis*] | 0.0 | 94.50 |
| COE11 | OP314711 | 560 | Yes | AIY68354.1 esterase [*Leptinotarsa decemlineata*] | 0.0 | 51.41 |
| COE12 | OP314712 | 555 | Yes | XP_023311611.1 venom carboxylesterase-6-like [*Anoplophora glabripennis*] | 0.0 | 77.70 |
| COE13 | OP314713 | 535 | Yes | XP_018568325.1 cholinesterase 1 [*Anoplophora glabripennis*] | 0.0 | 82.77 |
| COE14 | OP314714 | 562 | Yes | QYA71950.1 carboxylesterase [*Anoplophora glabripennis*] | 0.0 | 83.84 |
| COE15 | OP314715 | 466 | No | XP_018572491.1 venom carboxylesterase-6 isoform X1 [*Anoplophora glabripennis*] | 0.0 | 81.16 |
| COE16 | OP314716 | 592 | Yes | XP_018575847.1 esterase E4-like [*Anoplophora glabripennis*] | 0.0 | 81.27 |
| COE17 | OP314717 | 611 | Yes | XP_018565656.1 liver carboxylesterase 4-like [*Anoplophora glabripennis*] | 0.0 | 93.63 |
| COE18 | OP314718 | 556 | Yes | XP_023016385.1 venom carboxylesterase-6-like [*Leptinotarsa decemlineata*] | 0.0 | 68.35 |
| COE19 | OP314719 | 191 | No | XP_023311605.1 venom carboxylesterase-6-like [*Anoplophora glabripennis*] | 3e-86 | 70.17 |
| COE20 | OP314720 | 282 | No | XP_018563677.1 cholinesterase 1 [*Anoplophora glabripennis*] | 2e-159 | 80.14 |
| COE21 | OP314721 | 480 | No | XP_023310706.1 venom carboxylesterase-6 [*Anoplophora glabripennis*] | 0.0 | 87.71 |
| COE22 | OP314722 | 551 | Yes | QYA71944.1 carboxylesterase [*Anoplophora glabripennis*] | 0.0 | 80.58 |
| COE23 | OP314723 | 459 | Yes | XP_018570516.1 juvenile hormone epoxide hydrolase 1-like [*Anoplophora glabripennis*] | 0.0 | 81.72 |
| COE24 | OP314724 | 561 | Yes | XP_018567741.1 venom carboxylesterase-6 [*Anoplophora glabripennis*] | 0.0 | 87.61 |
| COE25 | OP314725 | 833 | Yes | XP_044266234.1 neuroligin-4, X-linked-like [*Tribolium madens*] | 0.0 | 83.67 |
| COE26 | OP314726 | 561 | Yes | XP_023018925.1 venom carboxylesterase-6-like [*Leptinotarsa decemlineata*] | 0.0 | 56.42 |
| COE27 | OP314727 | 554 | Yes | QYA71948.1 carboxylesterase [*Anoplophora glabripennis*] | 0.0 | 81.95 |
| COE28 | OP314728 | 557 | Yes | XP_023310181.1 cholinesterase 1 [*Anoplophora glabripennis*] | 0.0 | 91.22 |
| COE29 | OP314729 | 554 | Yes | XP_023310706.1 venom carboxylesterase-6 [*Anoplophora glabripennis*] | 0.0 | 89.22 |
| COE30 | OP314730 | 540 | Yes | XP_018563545.1 venom carboxylesterase-6-like [*Anoplophora glabripennis*] | 0.0 | 92.78 |
| COE31 | OP314731 | 546 | Yes | XP_018574555.1venom carboxylesterase-6-like [*Anoplophora glabripennis*] | 0.0 | 93.41 |
| COE32 | OP314732 | 563 | Yes | XP_023311239.1 cholinesterase 2-like [*Anoplophora glabripennis*] | 0.0 | 50.44 |
| COE33 | OP314733 | 342 | No | XP_018579839.1 neuroligin-1-like [*Anoplophora glabripennis*] | 0.0 | 95.91 |
| COE34 | OP314734 | 566 | Yes | QYA71946.1 carboxylesterase [*Anoplophora glabripennis*] | 0.0 | 89.22 |
| COE35 | OP314735 | 539 | Yes | XP_018562621.1 venom carboxylesterase-6-like [*Anoplophora glabripennis*] | 0.0 | 74.77 |
| COE36 | OP314736 | 315 | No | XP_018572491.1 venom carboxylesterase-6 isoform X1 [*Anoplophora glabripennis*] | 0.0 | 83.39 |
| COE37 | OP314737 | 554 | No | XP_018573086.1 venom carboxylesterase-6 [*Anoplophora glabripennis*] | 0.0 | 89.87 |
| COE38 | OP314738 | 334 | No | XP_018562621.1 venom carboxylesterase-6-like [*Anoplophora glabripennis*] | 5e-173 | 72.16 |
| COE39 | OP314739 | 550 | No | XP_018568320.1 cholinesterase 2-like isoform X2 [*Anoplophora glabripennis*] | 0.0 | 69.27 |
| COE40 | OP314740 | 408 | No | XP_023310399.1 cholinesterase 1 [*Anoplophora glabripennis*] | 0.0 | 73.53 |
| COE41 | OP314741 | 205 | No | XP_018563677.1 cholinesterase 1 [*Anoplophora glabripennis*] | 2e-101 | 73.66 |
| COE42 | OP314742 | 559 | Yes | XP_018572851.1 venom carboxylesterase-6 [*Anoplophora glabripennis*] | 0.0 | 79.61 |
| COE43 | OP314743 | 583 | Yes | XP_023309808.1 venom carboxylesterase-6-like isoform X5 [*Anoplophora glabripennis*] | 0.0 | 74.14 |
| COE44 | OP314744 | 557 | Yes | XP_023310181.1 cholinesterase 1 [*Anoplophora glabripennis*] | 0.0 | 68.82 |
| COE45 | OP314745 | 551 | Yes | XP_018565110.1 cholinesterase 2-like [*Anoplophora glabripennis*] | 0.0 | 89.29 |
| COE46 | OP314746 | 245 | No | XP_018575281.1 juvenile hormone esterase-like [*Anoplophora glabripennis*] | 5e-119 | 69.80 |
| COE47 | OP314747 | 534 | No | XP_018563677.1 cholinesterase 1 [*Anoplophora glabripennis*] | 0.0 | 73.73 |
| COE48 | OP314748 | 301 | No | XP_018563479.1 lipid droplet-associated hydrolase [*Anoplophora glabripennis*] | 0.0 | 87.09 |
| COE49 | OP314749 | 183 | No | XP_018562033.1 venom carboxylesterase-6 [*Anoplophora glabripennis*] | 2e-95 | 77.05 |
| COE50 | OP314750 | 601 | Yes | APO36904.1 juvenile hormone esterase [*Monochamus alternatus*] | 0.0 | 89.68 |
| COE51 | OP314751 | 567 | Yes | XP_018577068.1 venom carboxylesterase-6 [*Anoplophora glabripennis*] | 0.0 | 90.83 |
| COE52 | OP314752 | 349 | No | XP_023310400.1 cholinesterase-like [*Anoplophora glabripennis*] | 0.0 | 75.98 |
| COE53 | OP314753 | 564 | Yes | XP_023310404.1 cholinesterase 1-like isoform X2 [*Anoplophora glabripennis*] | 0.0 | 74.09 |
| COE54 | OP314754 | 288 | No | XP_018562621.1 venom carboxylesterase-6-like [*Anoplophora glabripennis*] | 1e-152 | 75.00 |
| COE55 | OP314755 | 568 | Yes | XP_018565293.1 venom carboxylesterase-6-like [*Anoplophora glabripennis*] | 0.0 | 83.92 |
| COE56 | OP314756 | 549 | Yes | XP_018562955.1 venom carboxylesterase-6-like [*Anoplophora glabripennis*] | 0.0 | 82.12 |
| COE57 | OP314757 | 598 | Yes | XP_018566534.1 carboxylesterase 5A [*Anoplophora glabripennis*] | 0.0 | 88.13 |
| COE58 | OP314758 | 555 | Yes | KAG5895360.1 hypothetical protein JTB14_029587 [*Gonioctena quinquepunctata*] | 0.0 | 52.17 |
| COE59 | OP314759 | 531 | Yes | QYA71945.1 carboxylesterase [*Anoplophora glabripennis*] | 0.0 | 80.04 |
| COE60 | OP314760 | 118 | No | XP_018573236.1 acetylcholinesterase [*Anoplophora glabripennis*] | 8e-75 | 96.61 |
| COE61 | OP314761 | 327 | No | XP_023313107.1 carboxylesterase 1D-like [*Anoplophora glabripennis*] | 0.0 | 72.82 |
| COE62 | OP314762 | 249 | No | XP_018570300.1 neuroligin-4, Y-linked-like, partial [*Anoplophora glabripennis*] | 9e-178 | 98.39 |
| COE63 | OP314763 | 106 | No | XP_023312043.1 esterase FE4-like [*Anoplophora glabripennis*] | 5e-54 | 79.44 |
| COE64 | OP314764 | 218 | No | XP_018573925.1 acyl-protein thioesterase 1 [*Anoplophora glabripennis*] | 6e-151 | 91.74 |
| COE65 | OP314765 | 567 | Yes | QYA71952.1 carboxylesterase [*Anoplophora glabripennis*] | 0.0 | 77.64 |
| COE66 | OP314766 | 546 | No | XP_018569636.1 esterase FE4-like [*Anoplophora glabripennis*] | 0.0 | 79.63 |
| COE67 | OP314767 | 150 | No | XP_023310412.1 LOW QUALITY PROTEIN: acetylcholinesterase-like [*Anoplophora glabripennis*] | 2e-78 | 79.33 |
| COE68 | OP314768 | 228 | No | XP_018579600.1 lysophospholipase-like protein 1 [*Anoplophora glabripennis*] | 1e-148 | 88.16 |
| COE69 | OP314769 | 401 | Yes | XP_018571264.1 protein phosphatase methylesterase 1 [*Anoplophora glabripennis*] | 0.0 | 94.51 |
| COE70 | OP314770 | 417 | No | AIY68369.1 esterase [*Leptinotarsa decemlineata*] | 1e-145 | 50.60 |
| COE71 | OP314771 | 368 | No | QYA71942.1 carboxylesterase [*Anoplophora glabripennis*] | 0.0 | 73.02 |
| COE72 | OP314772 | 325 | No | XP_018573236.1 acetylcholinesterase [*Anoplophora glabripennis*] | 0.0 | 96.00 |
| COE73 | OP314773 | 168 | No | XP_008201044.1 PREDICTED: acetylcholinesterase 2 isoform X1 [*Tribolium castaneum*] | 1e-101 | 90.53 |
| COE74 | OP314774 | 130 | No | ABX44678.1 acetylcholinesterase, partial [*Alphitobius diaperinus*] | 6e-85 | 91.54 |
| COE75 | OP314775 | 188 | No | XP_018568320.1 cholinesterase 2-like isoform X2 [*Anoplophora glabripennis*] | 4e-87 | 68.98 |
| COE76 | OP314776 | 108 | No | XP_018579806.1 neuroligin-4, Y-linked-like [*Anoplophora glabripennis*] | 2e-66 | 95.37 |
| COE77 | OP314777 | 136 | No | XP_018562603.1 neuroligin-3-like [*Anoplophora glabripennis*] | 3e-88 | 98.53 |
| **Glutathione-S-transferase (GST)** | | | | | | |
| GSTd1 | OP314778 | 216 | Yes | QYA72012.1 glutathione S-transferase [*Anoplophora glabripennis*] | 3e-113 | 71.50 |
| GSTd2 | OP314779 | 231 | Yes | XP_018564199.1 glutathione S-transferase 1-1 [*Anoplophora glabripennis*] | 8e-160 | 92.64 |
| GSTd3 | OP314780 | 217 | Yes | XP_023311254.1 glutathione S-transferase 1-1 [*Anoplophora glabripennis*] | 1e-121 | 78.80 |
| GSTd4 | OP314781 | 216 | Yes | XP_023311254.1 glutathione S-transferase 1-1 [*Anoplophora glabripennis*] | 2e-147 | 93.06 |
| GSTd5 | OP314782 | 190 | No | QYA72012.1 glutathione S-transferase [*Anoplophora glabripennis*] | 5e-111 | 80.53 |
| GSTd6 | OP314783 | 215 | Yes | XP_023311258.1 glutathione S-transferase 1-1 [*Anoplophora glabripennis*] | 2e-131 | 84.19 |
| GSTd7 | OP314784 | 241 | Yes | QYA72011.1 glutathione S-transferase [*Anoplophora glabripennis*] | 9e-158 | 86.72 |
| GSTd8 | OP314785 | 220 | Yes | XP_018568551.1 glutathione S-transferase 1 [*Anoplophora glabripennis*] | 8e-154 | 93.18 |
| GSTd9 | OP314786 | 164 | No | XP_018568551.1 glutathione S-transferase 1 [*Anoplophora glabripennis*] | 5e-83 | 62.73 |
| GSTd10 | OP314787 | 123 | No | QYA72012.1 glutathione S-transferase [*Anoplophora glabripennis*] | 4e-53 | 67.48 |
| GSTd11 | OP314788 | 219 | Yes | XP_023311254.1 glutathione S-transferase 1-1 [*Anoplophora glabripennis*] | 5e-135 | 84.26 |
| GSTe1 | OP314789 | 216 | Yes | XP_018576730.1 glutathione S-transferase 1 [*Anoplophora glabripennis*] | 3e-127 | 82.41 |
| GSTe2 | OP314790 | 216 | Yes | XP_018564051.1 glutathione S-transferase 1 [*Anoplophora glabripennis*] | 2e-134 | 85.65 |
| GSTe3 | OP314791 | 158 | No | QYA72005.1 glutathione S-transferase [*Anoplophora glabripennis*] | 3e-70 | 67.10 |
| GSTe4 | OP314792 | 214 | Yes | XP_018576731.1 glutathione S-transferase 1-like [*Anoplophora glabripennis*] | 1e-89 | 56.81 |
| GSTe5 | OP314793 | 216 | Yes | QYA72005.1 glutathione S-transferase [*Anoplophora glabripennis*] | 3e-133 | 82.87 |
| GSTe6 | OP314794 | 216 | Yes | XP_018564047.1 glutathione S-transferase 1 isoform X2 [*Anoplophora glabripennis*] | 3e-137 | 85.19 |
| GSTe7 | OP314795 | 216 | Yes | XP_018564046.1 glutathione S-transferase 1 isoform X1 [*Anoplophora glabripennis*] | 1e-126 | 80.09 |
| GSTe8 | OP314796 | 216 | Yes | XP_018564052.1 glutathione S-transferase 1-like [*Anoplophora glabripennis*] | 1e-143 | 90.28 |
| GSTe9 | OP314797 | 215 | Yes | XP_018564052.1 glutathione S-transferase 1-like [*Anoplophora glabripennis*] | 3e-120 | 76.64 |
| GSTo1 | OP314798 | 241 | Yes | QVK45137.1 GSTo2 [*Pagiophloeus tsushimanus*] | 7e-118 | 66.80 |
| GSTo2 | OP314799 | 244 | Yes | APX61043.1 putative glutathione S-transferase omega class member 4 [*Leptinotarsa decemlineata*] | 1e-140 | 79.10 |
| GSTs1 | OP314800 | 206 | Yes | XP_018560985.1 glutathione S-transferase [*Anoplophora glabripennis*] | 3e-111 | 73.79 |
| GSTs2 | OP314801 | 204 | Yes | XP_018573274.1 glutathione S-transferase [*Anoplophora glabripennis*] | 2e-143 | 94.12 |
| GSTs3 | OP314802 | 114 | No | XP_018560993.1 glutathione S-transferase-like isoform X1 [*Anoplophora glabripennis*] | 2e-49 | 74.14 |
| GSTs4 | OP314803 | 206 | Yes | XP_018560987.1 glutathione S-transferase [*Anoplophora glabripennis*] | 8e-117 | 75.49 |
| GSTs5 | OP314804 | 206 | Yes | XP_018560948.1 glutathione S-transferase-like [*Anoplophora glabripennis*] | 4e-117 | 80.60 |
| GSTs6 | OP314805 | 151 | No | XP_018560987.1 glutathione S-transferase [*Anoplophora glabripennis*] | 8e-81 | 76.16 |
| GSTs7 | OP314806 | 121 | No | XP_018560987.1 glutathione S-transferase [*Anoplophora glabripennis*] | 6e-64 | 76.03 |
| GSTt1 | OP314807 | 229 | Yes | XP_018576339.1 glutathione S-transferase theta-1-like [*Anoplophora glabripennis*] | 7e-143 | 83.84 |
| GSTt2 | OP314808 | 229 | Yes | XP_018576338.1 glutathione S-transferase theta-1 [*Anoplophora glabripennis*] | 7e-145 | 83.84 |
| GSTz1 | OP314809 | 212 | Yes | AIL23553.1 glutathione S-transferase zeta [*Tenebrio molitor*] | 4e-112 | 72.95 |

AA, amino acid. ORF, open reading frame. FL, full length.
